# Endothelial soluble APP/APLP2 promote heart repair through KIT-mediated angiogenesis

**DOI:** 10.1126/sciadv.aeh0301

**Published:** 2026-05-22

**Authors:** Haruya Kawase, Shangmin Liu, Sabrina Kurz, Verena Bengelsdorff, Remy Bonnavion, Young-June Jin, Kenneth Anthony Roquid, Haaglim Cho, Guozheng Liang, Marija Banićević, Niharika Shiva, Paula Sofía Yunes-Leites, Stefan Günther, Lukas Tombor, Stefanie Dimmeler, Mario Looso, Kenny Mattonet, Nina Wettschureck, Ulrike C. Müller, Stefan Offermanns

**Affiliations:** ^1^ Max Planck Institute for Heart and Lung Research, Department of Pharmacology, Ludwigstr. 43, 61231 Bad Nauheim, Germany.; ^2^ Department of Cardiology, Nagoya University School of Medicine, 65 Tsurumai-cho, Showa-ku, Nagoya, 466-8550, Japan.; ^3^ Department of Functional Genomics, Institute of Pharmacy and Molecular Biotechnology, Heidelberg University, Heidelberg, Germany.; ^4^ Cardiopulmonary Institute, Bad Nauheim, Germany.; ^5^ German Center for Cardiovascular Research, partner site Frankfurt/Rhine-Main, Bad Nauheim, Germany.; ^6^ Max Planck Institute for Heart and Lung Research, Deep Sequencing Platform, Ludwigstr. 43, 61231 Bad Nauheim, Germany.; ^7^ Institute for Cardiovascular Regeneration, Goethe University Frankfurt, Theodor-Stern-Kai 7, 60590 Frankfurt am Main, Germany.; ^8^ Max Planck Institute for Heart and Lung Research, Bioinformatics, Ludwigstr. 43, 61231 Bad Nauheim, Germany.; ^9^ Imaging Platform, Max Planck Institute for Heart and Lung Research, Ludwigstr. 43, 61231 Bad Nauheim, Germany.; ^10^ Center for Molecular Medicine, Goethe University Frankfurt, Theodor-Stern-Kai 7, 60590 Frankfurt, Germany.

## Abstract

Amyloid precursor protein (APP) gives rise to amyloid-β, a pathological factor in Alzheimer’s disease. However, the physiological role of APP and its homolog amyloid precursor–like protein 2 (APLP2), which are also widely expressed outside the nervous system, is largely unknown. Here, we show that endothelial APP and APLP2 are required for postischemia angiogenesis after myocardial infarction (MI). We found that hypoxia induced the endothelial expression of α-secretases, resulting in nonamyloidogenic processing of APP and APLP2 into the soluble forms APPsα and APLP2sα. Loss of endothelial APP and APLP2 led to decreased neovascularization as well as increased heart failure and mortality after MI, a phenotype that could be rescued by endothelial expression of APPsα. APPsα and APLP2sα exerted their proangiogenic effect by positive allosteric modulation of the endothelial receptor tyrosine kinase KIT, which promotes postischemia neovascularization. Our data identify a function of APP and APLP2 in endothelial cells, which is required for postischemia tissue repair, and suggest approaches to improve regeneration after MI and other ischemic diseases.

## Teaser

APPsα and APLP2sα are positive allosteric modulators of KIT and thereby promote neovascularization after myocardial infarction.

## INTRODUCTION

Acute myocardial infarction (MI) is one of the leading causes of mortality and morbidity worldwide (*1*). It is caused by acute coronary artery thrombosis, resulting in vessel occlusion, ischemia, and eventually necrosis of cardiac tissue. Due to the limited ability of the cardiac muscle to regenerate, a scar forms that may lead to cardiac failure (*2*, *3*). Cardiac repair after MI involves the angiogenic formation of new blood vessels in the border and infarct zone (*4*). Angiogenesis after MI in the infarct and border zone occurs by clonal expansion of preexisting resident endothelial cells (*5*–*7*). Improved blood perfusion provided by newly formed microvessels prevents cardiomyocyte death, reduces scar size, and improves cardiac function after MI and can be exploited therapeutically (*8*, *9*). Various studies had shown that vascular endothelial growth factor (VEGF) and fibroblast growth factor (FGF) promote postischemia neovascularization in preclinical models (*10*–*14*). However, multiple clinical trials delivering different forms of VEGF and FGF to the heart failed to show beneficial effects of these growth factors (*15*, *16*), indicating that the understanding of postinfarct angiogenesis is incomplete. The VEGFA–myocyte enhancer factor 2 pathway, which was thought to be involved in neovascularization in adult hearts after MI, appears to be rather impaired (*17*). Various immune cells including myeloid cells are recruited to the heart after MI and contribute to cardiac repair including angiogenesis (*18*–*20*), and myeloid cell–derived factors as well as the expansion of KIT-expressing endothelial cells have been shown to promote angiogenesis after MI (*21*, *22*). However, the molecular mechanisms involved in cardiac postischemia neovascularization still remain unclear.

## RESULTS

### Endothelial APP/APLP2 expression and in vitro function

**Fig. 1.**
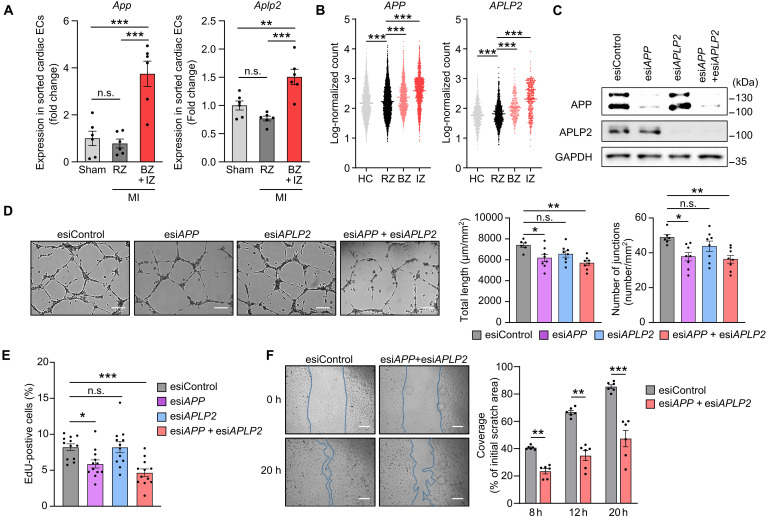
Expression and in vitro function of endothelial APP/APLP2. (**A**) Endothelial cells were isolated by fluorescence-activated cell sorting (FACS) from the infarct zone (IZ), border zone (BZ), or remote zone (RZ) of wild-type hearts 7 days after MI or from a corresponding region of sham operated mice (*n* = 6 mice per group), and expression levels of *App* and *Aplp2* were determined by qRT-PCR. ECs, endothelial cells. (**B**) RNA sequencing–derived expression levels of *APP* and *APLP2* in single cardiac endothelial cells from the indicated areas of the heart of patients with acute MI or of healthy control individuals (HC) (*90*). (**C** and **D**) Human umbilical vein endothelial cells (HUVECs) were transfected with control esiRNA or with esiRNAs directed against *APP* and/or *APLP2*, and efficiency of knockdown was validated by immunoblotting (C), or cells were seeded in Matrigel to study tube formation (D). Shown are representative images at 8 hours. The bar diagram shows the statistical evaluation of total tube length and number of junctions [*n* = 6 (control) and *n* = 8 (other conditions) independently performed experiments]. Scale bars, 200 μm. (**E**) Proliferation of HUVECs was analyzed by determining 5-ethynyl-2′-deoxyuridine (EdU) incorporation. 24 hours after the indicated knockdown, HUVECs were incubated with EdU (10 μM) for 12 hours, and the percentage of proliferating EdU-positive cells was determined (*n* = 12 wells). Scale bars, 200 μm. (**F**) Wound-healing assay was performed with HUVECs transfected with control esiRNA or esiRNA directed against *APP* and *APLP2*. Shown are representative images as well as the statistical analysis at the indicated time points after wounding the cell monolayer (*n* = 6 independently performed experiments). h, hours. Scale bars, 200 μm. Shown are means ± SEM; *P ≤ 0.05; **P ≤ 0.01; ***P ≤ 0.001; n.s., not significant [one-way ANOVA with Tukey’s (A, B, and E) or Šidák’s multiple comparisons test (D), and two-way ANOVA with Šidák’s multiple comparisons test (F)].

In an analysis of genes encoding transmembrane proteins and expressed in endothelial cells of mouse and human hearts, we noticed high expression levels of genes encoding amyloid precursor protein (APP), the source of amyloid-β peptide, and its close homolog amyloid precursor–like protein 2 (APLP2) (fig. S1, A to F) in contrast to APLP1 whose expression is restricted to the nervous system (*23*). We therefore determined expression of endothelial *App* and *Aplp2* in the mouse heart under pathological conditions such as MI. The relative expression of *App* and, to a lesser degree, of *Aplp2* in endothelial cells of the infarct and border zones further increased, whereas expression in the remote zone remained largely unchanged (Fig. 1A). Analysis of single-cell nucleus expression in the human heart after MI revealed also a significant increase in endothelial *APP* and *APLP2* expression in the border and infarct zones, whereas expression in the remote zone remained unchanged (Fig. 1B). Because APP has previously been linked to various in vitro functions of endothelial cells (*24*, *25*), we tested in human umbilical vein endothelial cells (HUVECs) the effect of a knockdown of APP alone or together with APLP2. Endothelial tube formation as well as proliferation of HUVECs was not affected by knockdown of APLP2 but was reduced after knockdown of APP and both APP and APLP2 (Fig. 1, C to E, and fig. S1G). Knockdown of APP and APLP2 also reduced the ability of endothelial cells to migrate (Fig. 1F).

### Increased mortality and impaired cardiac function after MI in mice with loss of endothelial APP and APLP2

**Fig. 2.**
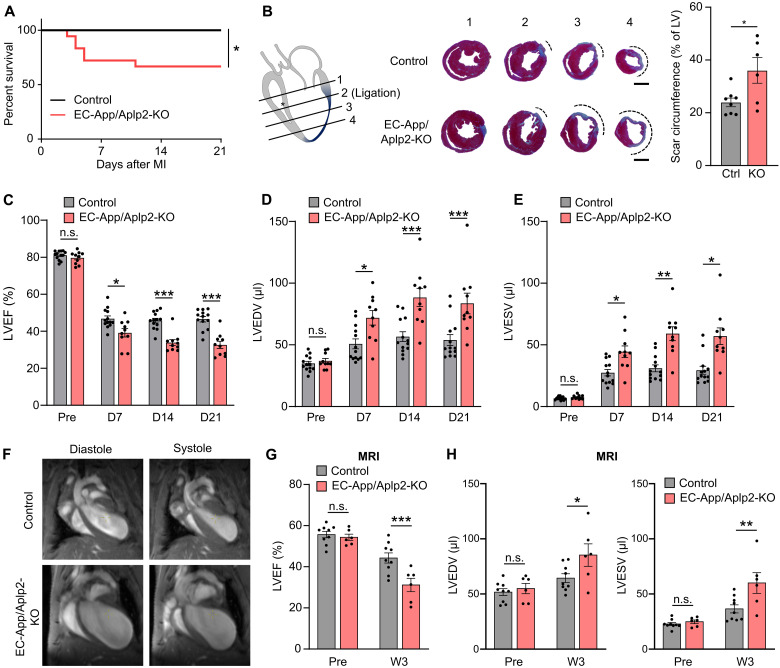
Endothelial loss of APP/APLP2 impairs cardiac function after MI. MI was induced by ligation of the left anterior descending (LAD) coronary artery in male control and EC-App/Aplp2-KO mice. (**A**) Survival of control (*n* = 13) and EC-App/Aplp2-KO mice (*n* = 18) after MI. (**B**) Scar size was determined after Masson-trichrome staining 21 days after MI. Serial sections were cut from the site of the ligation (section 2) toward the base and apex. Averaged scar circumference in the four indicated sections, which are separated by 800 μm each, was analyzed and the average is shown (*n* = 8 mice, control; *n* = 6 mice, EC-App/Aplp2-KO). Scale bars, 2 mm. (**C** to **E**) Cardiac function was determined before (Pre) and 1 to 3 weeks after MI in control (*n* = 13) and EC-App/Aplp2-KO mice (*n* = 10). LVEF, left ventricular ejection fraction (C); LVEDV, left ventricular end-diastolic volume (D); LVESV, left ventricular end-systolic volume (E). (**F** to **H**) Cardiac function was analyzed by magnetic resonance imaging (MRI) before (Pre) and 3 weeks after MI in control (*n* = 9) and EC-App/Aplp2-KO mice (*n* = 6). Representative images at diastole and systole are shown (F). The bar diagrams show the statistical evaluation of LVEF (G), LVEDV, and LVESV (H). D, day; W, week. Shown are means ± SEM; *P ≤ 0.05; **P ≤ 0.01; ***P ≤ 0.001; n.s., not significant [Gehan-Breslow-Wilcoxon test (A), unpaired two-tailed *t* test (B), and two-way repeated-measures ANOVA with Šidák’s multiple comparisons test (C to E, G, and H)].

To test the potential role of endothelial APP and APLP2 in MI, we generated inducible endothelium–specific APP/APLP2–double-deficient mice by crossing Cdh5-CreER^T2^ mice (*26*) with *App*^fl/fl^;*Aplp2*^fl/fl^ animals (*27*) (herein referred to as EC-App/Aplp2-KO) (fig. S2, A and B). When subjected to acute MI, more than a quarter of the EC-App/Aplp2-KO mice died within 3 weeks after MI due to rupture of the cardiac wall, in contrast to control animals, which did not show any lethality (Fig. 2A). The surviving EC-App/Aplp2-KO mice had larger infarct scars compared to control animals (Fig. 2B) and showed a more severe contractile dysfunction (Fig. 2C), increased left ventricular dilatation (Fig. 2, D and E), and decreased fractional area change (FAC) (fig. S2C) compared to control animals when analyzed by echocardiography or by magnetic resonance imaging (MRI) (Fig. 2, F to H). The impaired heart function after MI in male mice was not due to a difference in infarct size 1 day after MI and was also observed in female mice (fig. S2, D to H). Survival and cardiac function of endothelium-specific *App* or *Aplp2* single knockouts (EC-App-KO and EC-Aplp2-KO, respectively) was indistinguishable from that of control animals after MI (fig. S3, A to C). We therefore conclude that combined endothelial loss of APP and APLP2 results in reduced survival and reduced cardiac function after MI, indicating that the highly related APP and APLP2 proteins can compensate for each other in this endothelial function.

### Reduced postischemia angiogenesis in EC-App/Aplp2-KO mice

**Fig. 3.**
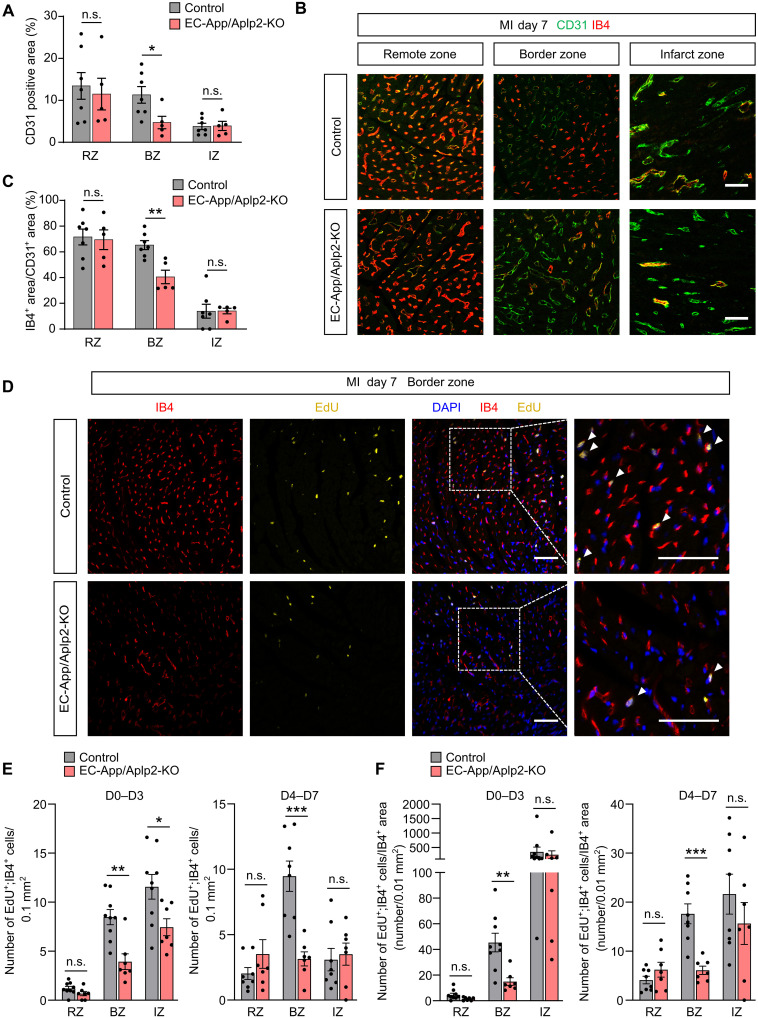
Reduced postischemia angiogenesis in EC-App/Aplp2-KO mice. (**A**) Shown is the CD31-positive area of histological sections from the infarct zone (IZ), border zone (BZ), and remote zone (RZ) 7 days after MI (*n* = 7 mice, control; *n* = 5 mice, EC-App/Aplp2-KO, three sections per mouse). (**B** and **C**) Seven days after MI, Alexa Fluor 647–IB4 was injected intravenously, and, 15 min later, hearts were collected from control and EC-App/Aplp2-KO mice. Shown are representative immunofluorescence confocal images of sections through the indicated areas stained with isolectin B4 (IB4) and an anti-CD31 antibody (B) as well as the statistical evaluation of the IB4^+^ area per CD31^+^ area (C) (*n* = 7 mice, control; *n* = 5 mice, EC-App/Aplp2-KO). (**D** to **F**) EdU was administrated to control and EC-App/Aplp2-KO mice via the drinking water for 3 days [day 0 (directly after MI) to day 3 or day 4 to day 7 after MI], and IB4 was injected intravenously 15 min before animals were euthanized to visualize perfused vessels. Shown are representative sections at the border zone stained for DAPI, EdU, and IB4 (D); the statistical evaluation of EdU^+^;IB4^+^ cells per area (E) [*n* = 9 mice, control (days 0 to 3); *n* = 7 mice, EC-App/Aplp2-KO (days 0 to 3); *n* = 8 mice, control (days 4 to 7); and *n* = 7 mice, EC-App/Aplp2-KO (days 4 to 7)]; and the statistical evaluation of EdU^+^;IB4^+^ cells per IB4^+^ area (F) in the infarct zone (IZ), border zone (BZ), and remote zone (RZ) [*n* = 9 mice (control, days 0 to 3); *n* = 7 mice (EC-App/Aplp2-KO, days 0 to 3); *n* = 8 mice (control, days 4 to 7); and *n* = 7 mice (EC-App/Aplp2-KO, days 4 to 7)]; at least three sections were analyzed per animal. Scale bars, 50 μm (B and D). Shown are means ± SEM; *P ≤ 0.05; **P ≤ 0.01; ***P ≤ 0.001; n.s., not significant [unpaired two-tailed *t* test or Mann-Whitney test based on normality test (A, C, E, and F)].

When we analyzed left ventricular wall cells by flow cytometry 3 days after MI, we could not find a difference in the number and activation of neutrophils, monocytes, macrophages, and T cells infiltrating the cardiac wall in the remote, border, or infarct area between control and EC-App/Aplp2-KO mice (fig. S4, A to D). However, we observed less endothelial cells in the border zones in EC-App/Aplp2-KO animals, whereas no changes in endothelial cell number were found in the remote zone (Fig. 3A). This was accompanied by a strongly reduced perfusion in the border zone 7 days after MI in EC-App/Aplp2-KO animals as determined by staining of endothelial cells with isolectin B4 (IB4), which had been injected intravenously before animals were euthanized (Fig. 3, B and C). To determine endothelial cell proliferation in different parts of the heart after MI, we treated mice for 3 days with 5-ethynyl-2′-deoxyuridine (EdU) either from day 0 to day 3 or from day 4 to day 7 after MI. In wild-type control animals, a considerable fraction of endothelial cells in the infarct and border zones had proliferated as indicated by EdU incorporation (Fig. 3, D to F). However, in EC-App/Aplp2-KO mice, proliferation of endothelial cells especially in the border zone between days 0 and 3 and between days 4 and 7 was strongly reduced compared to controls (Fig. 3, D to F). No differences in proliferating endothelial cells were found in the remote zone (Fig. 3, E and F). Thus, loss of endothelial APP/APLP2 results in a strong reduction of ischemia-induced neovascularization after MI, leading to reduced perfusion in the infarct and border zones, increased scar size, reduced left ventricular function, and increased postinfarct mortality.

### APPsα mediates the proangiogenic effect of endothelial APP

**Fig. 4.**
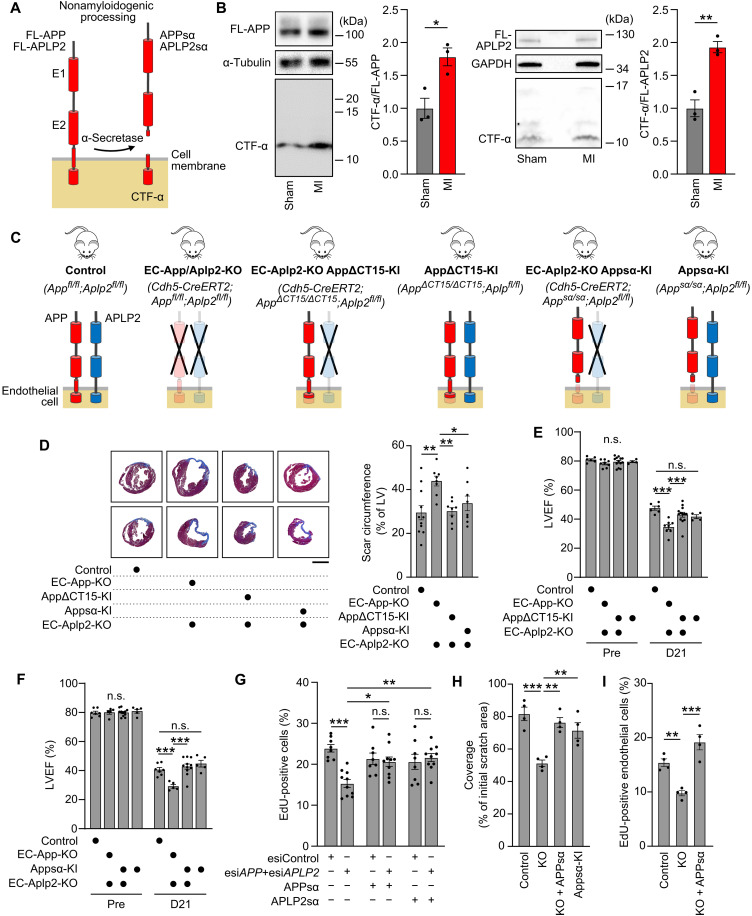
APPsα mediates proangiogenic APP effects. (**A**) Processing of APP and APLP2 by α-secretases. FL, full-length; CTF-α, C-terminal fragment-α. (**B**) Cleavage of APP and APLP2 in endothelial cells from border and infarct zones of hearts of wild-type mice 7 days after MI or from corresponding areas after sham operation were analyzed by immunoblotting with antibodies recognizing the C termini of APP and APLP2, respectively. Shown are relative levels of the processed CTF-α fragments compared to full-length APP and APLP2 (FL-APP/APLP2) (*n* = 3 independently performed experiments). (**C**) Schematic of tested mouse mutants. (**D**) Scar sizes 21 days after MI of the indicated mouse lines (*n* = 9 males and *n* = 2 females, control; *n* = 4 males and *n* = 4 females, EC-App/Aplp2-KO; *n* = 5 males and *n* = 3 females, EC-Aplp2-KO;AppΔCT15-KI; and *n* = 3 males and *n* = 5 females, EC-Aplp2-KO;Appsα-KI). Scale bar, 2 mm. (**E** and **F**) MI was induced in control (*n* = 3 males and 3 females), EC-App/Aplp2-KO (*n* = 4 males and 4 females), AppΔCT15-KI (*n* = 6 males and 7 females), and EC-Aplp2-KO;AppΔCT15-KI mice (*n* = 1 male and 3 females) (E) or in control (*n* = 5 males and 2 females), EC-App/Aplp2-KO (*n* = 4 males and 1 female), Appsα-KI (*n* = 3 males and 8 females), and EC-Aplp2-KO;Appsα-KI mice (*n* = 3 males and 2 females) (F). LVEF, left ventricular ejection fraction; Pre, before MI; D21, 21 days after MI. (**G**) Proliferation of HUVECs in the absence or presence of APPsα or APLP2sα was analyzed by determining EdU incorporation (*n* = 8 to 10 wells). (**H** and **I**) Wound-healing assay (H) and proliferation assay (I) using lung endothelial cells from control, EC-App/Aplp2-KO (KO), or EC-Aplp2-KO;Appsα-KI mice (Appsα-KI) mice (*n* = 4 mice per group) in the absence or presence of 20 nM APPsα. Shown are means ± SEM; *P ≤ 0.05; **P ≤ 0.01; ***P ≤ 0.001; n.s., not significant [two-way ANOVA with Šidák’s (B and G) or Tukey’s (E and F) multiple comparisons test, one-way ANOVA with Holm-Šidák’s multiple comparisons test (D), and one-way ANOVA with Dunnett’s multiple comparisons test (H and I)].

We then analyzed the full-length and cleaved forms of APP and APLP2 proteins in cardiac endothelial cells isolated after MI from the infarct and border zones and compared them with those of a corresponding area of healthy hearts. After MI, the nonamyloidogenic cleavage of APP and APLP2 was increased about twofold (Fig. 4, A and B, and fig. S5A). The increased processing of endothelial APP and APLP2 after MI was most likely due to increased cardiac expression of the α-secretase encoding genes *ADAM9*, *ADAM10*, and *ADAM17* in mice and humans after MI (fig. S5, B and C). This is supported by our observation that expression of *ADAM9*, *ADAM10*, and *ADAM17* in human cardiac microvascular endothelial cells is increased in response to hypoxia (fig. S5D).

Because the nonamyloidogenic processing of APP resulting in the formation of APP-secreted α (APPsα) is the predominant mode of proteolytic cleavage in cardiac endothelial cells and because EC-Aplp2-KO mice are indistinguishable from control animals, we tested whether APPsα is sufficient for the observed proangiogenic effect or whether membrane-bound APP might play a role. To this end, we used *App*^sα^ knock-in mice, which express no full-length APP but only APPsα. In addition, we also used *App*^ΔCT15^ knock-in mice that lack the last 15 C-terminal amino acids of APP, harboring a prominent protein interaction motif but still producing normal amounts of APPsα, while Aβ and APPsβ production is markedly reduced (*28*, *29*). When crossed with EC-Aplp2-KO mice (Fig. 4C), both knock-ins (herein referred to as Appsα-KI and AppΔCT15-KI, respectively) were able to rescue the phenotype of EC-App/Aplp2-KO mice and showed scar sizes (Fig. 4D) and cardiac functions comparable to those found in control animals (Fig. 4, E and F, and fig. S6, A to I). This indicates that the 15 C-terminal amino acids of APP, which have been shown to mediate downstream signaling of APP (*23*), are not required for postischemia neovascularization, whereas the expression of APPsα alone is sufficient to support postischemia neovascularization after MI. The critical role of endothelial APPsα in the postischemia response of the heart was confirmed by expression of APPsα in endothelial cells in vivo using adeno-associated virus 2 (AAV2)–QuadYF, which in combination with the *Tie1* promoter allows for endothelium specific transduction (fig. S7, A and B) (*30*, *31*) and which also rescued the phenotype of EC-App/Aplp2-KO mice (fig. S7, C to F).

Also, in vitro APPsα was able to rescue the effect of an APP/APLP2 knockdown in endothelial cells, which showed in the presence of APPsα normal proliferation and tube-forming ability (Fig. 4G and fig. S7G). Similarly, APPsα also rescued the defect in wound healing and proliferation of endothelial cells isolated from EC-App/Aplp2-KO mice as did the *App*^sα^ knock-in (Appsα-KI) (Fig. 4, H and I). Also, APLP2sα, which is highly homologous to APPsα, rescued the effect of an APP/APLP2 knockdown (Fig. 4G and fig. S7H). Thus, APPsα and APLP2sα appear to be responsible for the APP- and APLP2-dependent endothelial functions in vitro and for promoting postischemia neovascularization in vivo. Their effects occurred in the absence of full-length APP/APLP2 in endothelial cells, indicating that APPsα and APLP2sα induced their effects independently of full-length endogenous APP/APLP2 in an auto- or paracrine manner.

### APPsα induces endothelial effects through the receptor tyrosine kinase KIT

**Fig. 5.**
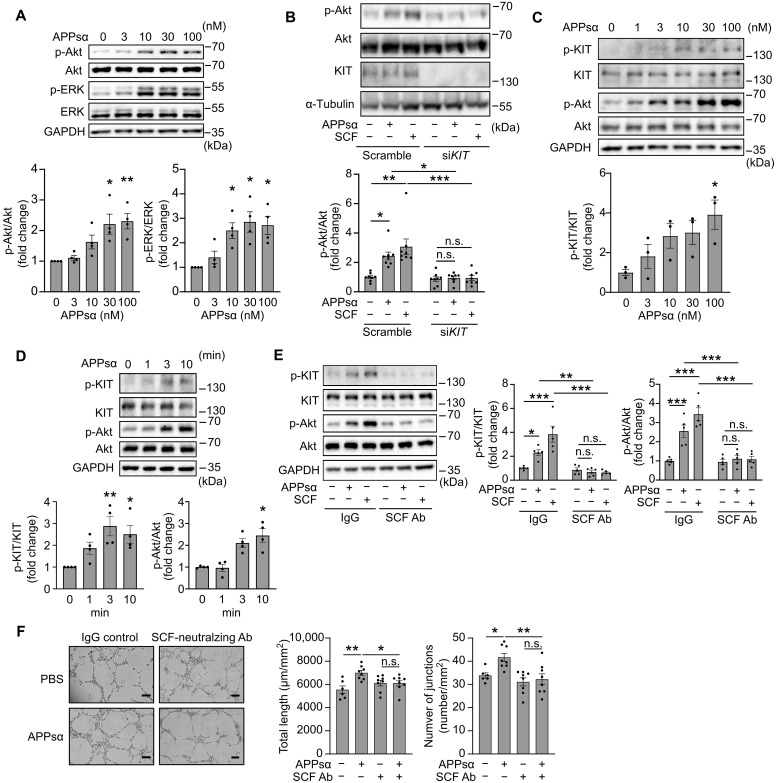
APPsα induces endothelial effects in a KIT-dependent manner. (**A**) HUVECs after esiRNA-mediated knockdown of APP and APLP2 were treated for 10 min with increasing concentration of APPsα, and phosphorylation of Akt (serine-473) and extracellular signal–regulated kinase (ERK; threonine-202/tyrosine-204) was analyzed by immunoblotting (*n* = 4). (**B**) HUVECs transfected with control small interfering RNA (siRNA) or siRNA directed against *KIT* were treated with APPsα (20 nM) or stem cell factor (SCF; 2.5 nM) for 10 min, and phosphorylation of KIT (tyrosine-568/570) and Akt was analyzed by immunoblotting (*n* = 8). (**C**) HUVECs were treated with the indicated concentrations of APPsα for 10 min, and phosphorylation of KIT and Akt was examined by immunoblotting (*n* = 3). (**D**) HUVECs were treated with APPsα (20 nM) for the indicated time periods, and the effects on KIT and Akt phosphorylation were examined by immunoblotting (*n* = 4). (**E**) HUVECs were pretreated with SCF-neutralizing antibody (Ab) or control immunoglobulin G (IgG) for 2 hours, followed by stimulation with APPsα (20 nM) or SCF (2.5 nM). The effects on KIT and Akt phosphorylation were examined by immunoblotting (*n* = 5). Shown is a representative of three to eight independently performed experiments and the statistical analysis (A to E). (**F**) HUVECs transfected with esiRNA against *APP* and *APLP2* were seeded in Matrigel to study tube formation in absence or presence of SCF-neutralizing antibody or control IgG and APPsα (20 nM). Shown are representative images at 8 hours. The bar diagram shows the statistical evaluation of total tube length and number of junctions [*n* = 6 (control, PBS) or *n* = 8 (all other conditions)]. Scale bars, 200 μm. Shown are means ± SEM; *P ≤ 0.05; **P ≤ 0.01; ***P ≤ 0.001; n.s., not significant [Kruskal-Wallis test with Dunn’s multiple comparisons test (A to D) and one-way ANOVA with Šidák’s multiple comparisons test (E and F)].

We next tested whether purified APPsα induced typical growth factor–induced signaling processes in endothelial cells in vitro. In HUVECs with knockdown of APP/APLP2, purified APPsα induced phosphorylation of Akt as well as of extracellular signal–regulated kinase (ERK) with maximal effects at a concentration of 30 nM (Fig. 5A). Given that APPsα induced downstream signaling events in HUVECs, which are typical for growth factor receptor–mediated responses, we tested the potential involvement of VEGFR2, FGFR1, epidermal growth factor (EGF) receptor (EGFR), and KIT, which have previously been reported to be involved in angiogenesis after MI (*21*, *32*–*34*). In contrast to VEGF, APPsα did not lead to phosphorylation of VEGFR2 at tyrosine-1175, indicative of VEGF receptor autophosphorylation and activation (fig. S8A). In addition, blockade of VEGFR2 signaling by the tyrosine kinase inhibitor SU5416 did not affect APPsα-induced Akt phosphorylation, whereas it blocked VEGF effects (fig. S8A). Also, knockdown of FGFR1 or inhibition of the EGFR by gefitinib did not affect APPsα-induced cellular signaling in HUVECs while it blocked the effects of FGF2 and EGF, respectively (fig. S8, B and C). However, when we tested the potential involvement of KIT, we found that knockdown of KIT blocks not only stem cell factor (SCF)–induced phosphorylation of Akt but also Akt phosphorylation induced by APPsα (Fig. 5B). APPsα induced KIT receptor phosphorylation and downstream signaling in a dose- and time-dependent manner (Fig. 5, C and D). Also, APLP2sα was able to induce KIT phosphorylation in endothelial cells (fig. S8D). Because endothelial cells produce and secrete SCF, the canonical ligand of KIT (fig. S8, E and F) (*35*, *36*), we tested the effect of an SCF-neutralizing antibody on APPsα-induced phosphorylation of KIT and Akt. Not only SCF-induced but also APPsα-induced KIT phosphorylation was blocked by scavenging of SCF (Fig. 5E). Similarly, the SCF-neutralizing antibody also blocked APPsα-induced tube formation (Fig. 5F). Consistent with a role of SCF/KIT in APPsα-induced postischemia angiogenesis, endothelial expression of *Kit* in the murine infarct zone and of *KIT* and *KITL*, which encodes SCF, in the human infarct zone is increased (fig. S8, G and H). These data show that APPsα-induced downstream signaling in endothelial cells in vitro is dependent on KIT as well as on the KIT ligand SCF.

### APPsα is a positive allosteric modulator of KIT

**Fig. 6.**
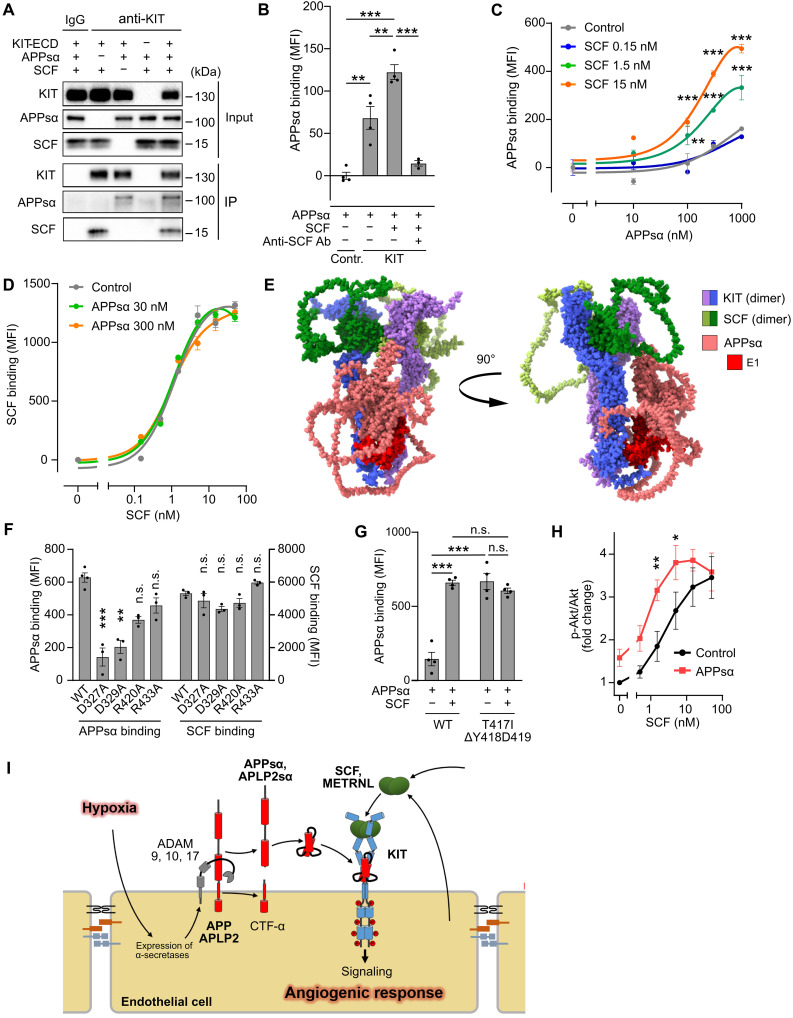
APPsα is a positive allosteric modulator of KIT. (**A**) Extracellular part of human KIT (KIT-ECD), APPsα, and SCF were incubated in different combinations. After immunoprecipition of KIT, immunoprecipitates (IP) were analyzed by immunoblotting using indicated antibodies. (**B**) Control HEK-293T cells (Contr.) or HEK-293T cells expressing KIT were incubated in the absence or presence of SCF-neutralizing antibody (anti-SCF Ab), SCF (5 nM), and His-tagged APPsα (1 μM), and binding of APPsα was determined by flow cytometry. Shown is the mean fluorescence intensity (MFI) of the His signal (*n* = 4). (**C**) Binding of His-tagged APPsα to KIT expressing HEK-293T cells in the absence or presence of the indicated SCF concentrations (*n* = 3). (**D**) Binding of biotinylated SCF to KIT expressing HEK-293T cells in the absence or presence of the indicated concentrations of APPsα was measured by flow cytometry after staining of cells with PerCP-conjugated streptavidin. Shown is the MFI of the streptavidin signal (*n* = 4). (**E**) Predicted structure of the multimer consisting of dimerized KIT, dimerized SCF, and APPsα by AlphaFold (*92*). (**F**) Binding of biotinylated SCF or His-tagged APPsα to wild-type (WT) KIT or the indicated KIT mutants expressed in HEK-293T cells (*n* = 3). (**G**) Binding of His-tagged APPsα to HEK-293T cells expressing wild-type (WT) KIT or the KIT mutant (T417I, Δ418-419) in the absence or presence of 10 nM SCF (*n* = 3). (**H**) HUVECs were treated with increasing concentrations of SCF in the absence and presence of 50 nM APPsα, and Akt phosphorylation was determined (*n* = 7). (**I**) Schematic representation of the role of APP and APLP2 in mediating neovascularization after MI. Shown are means ± SEM; *P ≤ 0.05; **P ≤ 0.01; ***P ≤ 0.001; n.s., not significant [one-way ANOVA with Šidák’s multiple comparisons test (B and F), two-way ANOVA with Šidák’s (C and G), or Tukey’s multiple comparisons test (H)].

We then tested whether recombinant APPsα is able to interact with the extracellular part of KIT. In coimmunoprecipitation experiments using recombinant proteins, we found in immunoprecipitates of anti-KIT antibodies not only KIT but also small amounts of APPsα (Fig. 6A), and the amount of APPsα precipitated by anti-KIT antibodies increased when SCF was present (Fig. 6A), indicating that KIT and APPsα may directly interact. To analyze the interaction of APPsα/APLP2sα and KIT in more detail, we performed binding experiments. APPsα as well as APLP2sα bound to KIT-expressing human embryonic kidney (HEK) 293T cells, but not to control cells, and binding was further increased by SCF (Fig. 6B and fig. S9A) but was abrogated in the presence of an SCF-neutralizing antibody (Fig. 6B). Similarly, APPsα binding to HUVEC was abrogated by knockdown of KIT, was increased by SCF, and was strongly reduced when an SCF-neutralizing antibody was present (fig. S9B). SCF concentration-dependently increased the maximal binding of APPsα to KIT (Fig. 6C). Also, meteorin-like (METRNL), which functions as a KIT agonist (*21*), increased APPsα binding to KIT (fig. S9C). In contrast, APPsα had no effect on SCF binding to KIT (Fig. 6D) but slightly increased binding of METRNL (fig. S9D). Because coimmunoprecipitation of KIT and APPsα as well as binding of APPsα and APLP2sα to KIT was increased by SCF and because SCF leads to dimerization of KIT (*37*), we hypothesized that APPsα and APLP2sα preferentially bind to KIT dimers. Consistent with this hypothesis, an AlphaFold2 in silico analysis of the interaction between the KIT extracellular domain and APPsα as well as APLP2sα showed a high interface predicted template modeling (ipTM) score for the KIT dimer and APPsα as well as APLP2sα (Fig. 6E and fig. S9, E and F), whereas the score for KIT monomers and APPsα/APLP2sα was much lower (fig. S9E). The interaction of APPsα/APLP2sα and the KIT dimer was predicted to involve the extracellular immunoglobulin–like domains 4 and 5 (D4 and D5, respectively) of KIT and the E1 and extension domains of APPsα and APLP2sα (Fig. 6E and fig. S9F). To test this model, we mutated four amino acids of KIT, which were predicted to mediate the interaction between APPsα/APLP2sα and KIT. While all four mutants still bound SCF (Fig. 6F), two mutants (D327A and E329A) showed a strongly reduced ability to bind APPsα (Fig. 6F). To experimentally test whether APPsα preferentially bound to KIT dimers, we studied binding of APPsα to the KIT(T417I, Δ418-419) mutant, which has been shown to lead to constitutive and SCF-independent KIT dimerization and KIT downstream signaling (*38*, *39*). Whereas binding of APPsα to wild-type KIT was increased by SCF, which induces KIT dimerization (Fig. 6G), basal binding of APPsα to the autodimerizing KIT(T417I, Δ418-419) mutant was increased and could not be further increased by SCF (Fig. 6G). Last, we tested how APPsα affected the effect of increasing concentrations of SCF on KIT phosphorylation and KIT downstream signaling. The data show that APPsα increased the potency but not efficacy of SCF both with regard to KIT-mediated Akt and ERK phosphorylation (Fig. 6H and fig. S9, G and H). Thus, APPsα functions as a positive allosteric modulator of KIT by binding to the D4 and D5 domains of KIT dimers and thereby promotes KIT activation and downstream signaling (Fig. 6I).

## DISCUSSION

APP is best known for its role in Alzheimer’s disease as it is the source of amyloid-β peptide, which accumulates in brain senile plaques (*40*–*43*). Much less is known about the physiological function of APP in the nervous system, where it has been shown to play roles in synaptogenesis, synaptic plasticity, regulation of neuronal network activity, and cognition as well as in neuroprotection (*23*, *44*–*50*). APP belongs to a family of transmembrane proteins that in addition consist of the APP-like proteins APLP1 and APLP2. In contrast to APLP1, which is expressed in a nervous system-specific manner, APP and APLP2 are ubiquitously expressed (*23*), but their function outside the nervous system is largely unknown. Here, we show that APP and APLP2 are highly expressed in cardiac endothelial cells, that their expression is further up-regulated in the border and infarct zones after MI, and that APP/APLP2 are required for postischemic neovascularization. Loss of endothelial APP and APLP2 results in reduced cardiac repair after MI in male and female mice. We show that this function is mediated by the soluble forms of APP/APLP2 resulting from nonamyloidogenic processing and that APPsα and APLP2sα promote the angiogenic response after MI by positive allosteric modulation of KIT in endothelial cells.

Our in vitro and in vivo data indicate that APP and APLP2 are required for the full postischemia angiogenic response. APLP2, which lacks the amyloid-β–peptide region, otherwise closely resembles APP with regard to domain structure (*23*), and its processing by secretases is very similar to that of APP (*51*–*53*). We show that the postischemic neovascularization is mediated by the large soluble ectodomains of APP and APLP2 released from endothelial cells. Consistent with a central role of endothelial APPsα/APLP2sα, we found in mice and humans an up-regulation of the endothelial expression of *ADAM9*, *ADAM10*, and *ADAM17* in the infarct and border zones after MI. This is consistent with data showing that expression of *ADAM9*, *ADAM10*, and *ADAM17* is induced by hypoxia mainly through hypoxia-inducible factor–1α (*54*–*57*), an effect that we also observed in cardiac microvascular endothelial cells in vitro.

The receptor tyrosine kinase KIT and its ligand SCF regulate proliferation, differentiation, and survival of cells including hematopoietic stem cells, germ cells, and melanocytes (*37*). Both SCF and KIT are widely expressed in peripheral endothelial cells during development and in the adult mammalian organism (*58*), and in vitro studies suggested a role in the regulation of angiogenesis (*59*). KIT is not required for physiological organ vascularization in the embryo (*60*) but modulates pathological retinal neovascularization (*61*). In the heart, endothelial cells are the major KIT-expressing cell type (*62*, *63*). Recently, Reboll *et al.* (*21*) showed that endothelial KIT mediates postinfarction neovascularization in the heart by expanding the KIT-expressing endothelial cell population in the infarct and border zone. The expansion of KIT-expressing mouse endothelial cells could be stimulated not only by SCF but also by METRNL, which is released from myeloid cells recruited to the heart after acute injury (*21*). Our data show that binding of METRNL and the activity of SCF are potentiated by APPsα/APLP2sα and that APPsα/APLP2sα are required for a full postischemia neovascularization response.

KIT is a member of the type III subfamily of receptor tyrosine kinases, which have a common activation mechanism (*64*). Binding of preexisting SCF dimers induces KIT dimerization, which allows homotypic contacts between the two extracellular membrane-proximal D4 and D5 domains of KIT (Fig. 6I). This interaction allows trans-autophosphorylation of KIT dimers, which leads to stimulation of tyrosine kinase activity and recruitment and activation of intracellular signaling molecules (*65*). Our data show that APPsα allosterically binds to the D4 and D5 domains of KIT dimers and thereby promotes activation of KIT by the orthosteric ligand SCF. APPsα/APLP2sα alone were not able to activate KIT but increased the potency of SCF. This was not due to an increased affinity of KIT for SCF but to an increased efficacy of the full agonist SCF. Thus, APPsα/APLP2sα function as positive allosteric modulators of KIT.

Multiple pieces of evidence indicate that APP has protective effects in brain ischemia and traumatic brain injury, which are mediated by APPsα (*23*, *45*). Mice with global APP deficiency show increased damage after traumatic brain injury (*66*, *67*), and this protective effect of APP was shown to be mediated by APPsα (*66*–*68*). Similarly, brain ischemia leads to increased mortality in global APP-deficient mice (*69*), and postischemic administration of APPsα or overexpression of APP under the control of the prion promoter, which also leads to expression in brain endothelial cells (*70*), protects against neuronal damage after ischemic injury and decreases the infarct size after brain ischemia (*71*, *72*). Although these beneficial effects of APPsα in brain ischemia and traumatic brain injury are believed to be due to direct effects on neurons, the experimental design of the studies would also be consistent with a role of APPsα in acting on endothelial cells in the brain and thereby promoting neural protection and neuroregeneration. Future work will have to test a role of APPsα/APLP2sα in the angiogenic response to ischemia in other tissues or organs, including the brain.

In conclusion, we demonstrate that APP and APLP2 play a pivotal role in postischemia neovascularization of the heart. Mechanistically, we show that this involves the nonamyloidogenically processed soluble ectodomain fragments of endothelial APP and APLP2, which function as positive allosteric modulators of the endothelial KIT receptor kinase. Our findings identify a critical function of APP and APLP2 outside the nervous system and highlight a central mechanism of cardiac tissue repair which may be targeted therapeutically.

## MATERIALS AND METHODS

### Reagents

Recombinant human SCF (catalog no. 300-07-50) and human VEGF165a (catalog no. 100-20-10) were from PeproTech. Human FGF-2 (catalog no. SRP4037-50UG), recombinant human His-tagged APPsα (catalog no. S9564), and SU5416 (catalog no. S8442-5MG) were from Sigma-Aldrich. Biotinylated human SCF was from ARCOBiosystems (catalog no. SCF-H82E1). Human EGF (catalog no. 72528S) and gefitinib (catalog no. 4765S) were from Cell Signaling Technology. Recombinant human KIT extracellular domain (1-516) (catalog no. 11996-H02H) was from Sino Biological. Human glutathione *S*-transferase (GST)–tagged METRNL was from Creative Biomart (catalog no. METRNL-3928H). Mouse SCF (catalog no. R88632) and 4′,6-diamidino-2-phenylindole (DAPI; catalog no. D3571) were from Invitrogen.

### Cell culture

HUVECs were purchased from Gibco, and human cardiac microvascular endothelial cells (HCMVECs) were from Lonza. Cells were cultured in EGM-2 medium (Lonza, catalog no. CC-3162) containing supplements (catalog no. CC-4176 or CC-4147) on plates coated with collagen (Corning Collagen I, rat tail; catalog no. 356236) at a final concentration of 40 μg/ml and were used for a maximal number of six passages. For stimulation, cells were serum starved for 2 hours. In some cases, cells were pretreated with SCF-neutralizing antibody (0.3 μg/ml; R&D Systems, catalog no. AF-255-NA), SU5416 (5 μM), or gefitinib (10 μM) for 2 hours before stimulation. When treated with APPsα, cells were transfected with endoribonuclease prepared siRNA (esiRNA) directed against *APP* and *APLP2* 48 hours before. HEK-293T cells were obtained from American Type Culture Collection. Cells were maintained in Dulbecco’s modified Eagle’s medium (DMEM) supplemented with 10% (v/v) fetal calf serum (FCS; Gibco, catalog no. 10270106), 2 mM l-glutamine (Thermo Fisher Scientific, catalog no. 25030123), and penicillin/streptomycin (PenStrep; 5 U/ml; Gibco, catalog no. 15140122). Mouse cardiac microvascular endothelial cells were obtained from Cell Biologics and maintained in complete endothelial cell medium (Cell Biologics, catalog no. PB-M1168). Cells were incubated at 37°C in a humidified atmosphere with 5% CO_2_. In experiments using HCMVECs under hypoxic conditions, cells were cultured in a hypoxia chamber in 1% O_2_ and 5% CO_2_ at 37°C.

### siRNA-mediated knockdown

Cells at 50 to 70% confluence were transfected with 28.5 nM small interfering RNA (siRNA) or esiRNA using Opti-MEM and Lipofectamine RNAiMAX (Invitrogen) as described previously (*73*). esiRNA directed against enhanced green fluorescent protein was used as a negative control. esiRNAs directed against *APP*, *APLP2*, and *EGFP* were from Sigma-Aldrich. The targeted sequences of esiRNAs used in this study are shown in table S1. Predesigned siRNAs targeting human *KIT* and human *FGFR1* were purchased from Sigma-Aldrich. To target human *KIT*, two siRNAs were combined. Their target sequences are shown in table S2. siRNA Universal Negative Control #1 (Sigma-Aldrich, catalog no. SIC001) was used as a control.

### Cloning and purification of recombinant murine APLP2sα

For expression in HEK-293T cells, the murine APLP2sα isoform c (APLP2_695_; NCBI Ref: NM_009691.3) was synthesized (BioCat, Germany) terminating at the α-secretase cleavage site at Arg^572^ (*53*). The sequence was cloned into a pcDNA3.1 plasmid and contained an N-terminal APP signal peptide, followed by 6× His-tag, a Tobacco Etch Virus (TEV) site, and 2× hemagglutinin (HA)–tag. The sequence was assembled via overlap extension polymerase chain reaction (PCR)–based HiFi Assembly (New England Biolabs, USA) using Bam HI/Eco RI restriction sites provided by the PCR primers for insertion into the vector backbone. HEK-293T cells were cultured in T175 flasks (Sarstedt, Germany) with DMEM (high glucose, GlutaMAX, Life Technologies, USA) supplemented with 10% FCS, 2 mM l-glutamine, and PenStrep (100 U/ml; Life Technologies, USA). The day before transfection, 1.6 × 10^7^ cells were seeded per flask. For transfection, 35 μg of plasmid DNA was mixed with 2.5 ml of DMEM, while 140 μl of 18 mM polyethylenimine (Sigma-Aldrich, USA) was mixed with 2.2 ml of DMEM, then combined with the DNA solution, and vortexed thoroughly. After a 20-min incubation at room temperature, the complex was added to the cells and incubated overnight at 37°C with 5% CO_2_. The next day, the medium was replaced with DMEM containing 2% FCS, 2 mM l-glutamine, and PenStrep (100 U/ml).

Supernatants were collected 48 to 72 hours posttransfection, centrifuged at 4000*g* for 30 min at 4°C to remove detached cells, and sterile filtered using a 0.22-μm filter cup (Merck Millipore, Germany). HisTrap TALON columns (Cytiva, 28-9537-66, USA) were used for affinity purification, with elution in 3 ml of buffer [50 mM NaPO_4_, 300 mM NaCl, and 150 mM imidazole (pH 7.4)]. Eluates were desalted using PD-10 columns (Cytiva, USA), eluted in Dulbecco’s phosphate-buffered saline (PBS; Life Technologies, USA), and concentrated with a VivaspinR20 (30-kDa molecular weight cutoff) centrifugal concentrator (Sigma-Aldrich, USA), following the manufacturer’s instructions. Protein concentration was measured using the Bicinchoninic Acid Assay (Sigma-Aldrich, USA). For purity and integrity assessment, 1 μg of purified protein was mixed with Laemmli buffer [0.04% bromophenol blue, 40% glycerol, 8% SDS, 250 mM tris-HCl (pH 6.8), and 20% β-mercaptoethanol] and separated on a 4% stacking/12% running tris-glycine SDS–polyacrylamide gel electrophoresis (PAGE) gel. Western blotting was performed as described by Baltissen *et al.* (*74*). The purity of the recombinant protein was confirmed using Bio-Rad’s stain-free method (*75*, *76*) with the ChemiDoc MP system (Bio-Rad, USA). Integrity of the recombinant protein was verified via anti-His, anti-HA, and APLP2-specific antibodies (see table S3). Stock solutions were aliquoted at 100 mM and stored at −80°C.

### Tube formation assay

Tube formation assay was performed to evaluate in vitro angiogenesis. Prethawed Matrigel (Corning BV, catalog no. 356231) was applied to a μ-Slide Angiogenesis well (IBIDI, catalog no. 81506) according to the manufacturer’s instructions. HUVECs transfected with the indicated esiRNA were then seeded onto the Matrigel, and images were captured using an Olympus IX81 live cell imaging system (10× objective lens) at 37°C in an environmental chamber with humidified atmosphere and 5% CO_2_. Analysis of tube formation was performed by using Angiogenesis Analyzer, a plugin installed in the ImageJ software (Fiji, version 2.14.0).

### Determination of cell proliferation

Twenty-four hours after transfection with the indicated esiRNA, HUVECs were incubated with EdU (10 μM) for 12 hours, and the percentage of proliferating EdU-positive cells were determined by the Click-iT Plus EdU Cell Proliferation Kit (Invitrogen, catalog no. C10635). Where indicated, cells were treated with APPsα or APLP2sα (20 nM). Mouse lung endothelial cells (MLECs) were incubated with EdU (10 μM) for 12 hours, and the percentage of proliferating EdU-positive endothelial (CD31-positive) cells was determined as described above.

### Wound-healing assay

HUVECs were transfected with control esiRNA or esiRNA directed against *APP* and *APLP2* and grown to confluence. Cells were scratched with the edge of a sterile 200-μl pipette tip to create a linear wound. Detached cells were gently washed away, and images of the wound area were captured at the indicated time points using a live-cell imaging microscope. The wound closure was quantified by measuring the area of the cell-free gap, and the percentage of wound closure was calculated relative to the initial wound area. MLECs were grown to confluence, and the assay was performed as described above. Where indicated, cells were treated with APPsα (20 nM).

### Western blotting

Samples were lysed in radioimmunoprecipitation assay buffer [25 mM tris-HCl (pH 7.6), 150 mM NaCl, 1% NP-40, 1% sodium deoxycholate, and 0.1% SDS] (Thermo Fisher Scientific, catalog no. 89900) supplemented with protease and phosphatase inhibitors (Thermo Fisher Scientific, catalog no. 81506). Proteins were separated by SDS-PAGE (tris-glycine gels with tris/glycine/SDS buffer) and transferred onto nitrocellulose membranes (Whatman, Dassel, Germany), using the Mini Trans-Blot Cell (Bio-Rad). Membranes were probed over night with specific primary antibodies as indicated and then with peroxidase-conjugated anti-rabbit or anti-mouse secondary antibodies (Cell Signaling Technology, catalog nos. 7074S and 7076S, respectively; 1:10,000) for 1 hour at room temperature. Bound antibodies were visualized by enhanced chemiluminescence reagent (Merck Millipore) and a ChemiDoc MP Imaging System (Bio-Rad). For analysis of APLP2 cleavage, samples were processed as described above, and blot membranes were blocked for 1 hour at room temperature in 3% (w/v) nonfat dry milk in PBS containing 0.05% Tween 20 and incubated overnight at 4°C with anti-APLP2 antibody (Synaptic Systems, catalog no. 490 011, 1:1000). Membranes were washed and incubated with horseradish peroxidase–conjugated secondary antibody (Proteintech, catalog no. RGAM001; 1:10,000) for 90 min at room temperature. Immunoreactive signals were detected using SignalFire Elite ECL Reagent (Cell Signaling Technology, catalog no. 12757).

Quantification of band intensities was analyzed by ImageJ software (Fiji, version 2.14.0). Equal loading was assessed using antibodies to glyceraldehyde-3-phosphate dehydrogenase or α-tubulin. Antibodies used for Western blotting are shown in table S3.

### Coimmunoprecipitation

Recombinant human KIT (0.2 μg), human SCF (0.2 μg), and human APPsα (0.3 μg) were coincubated in 800 μl of immunoprecipitation buffer [150 mM NaCl, 25 mM tris-HCl (pH 7.4), 1 mM EDTA, 1% NP-40, and 5% glycerol] supplemented with protease inhibitor cocktail (Roche, catalog no. 4693159001) together with anti-KIT antibodies (clone D13A2; Cell Signaling Technology, catalog no. 3074; 1:50) or immunoglobulin G isotype control (clone DA1E; Cell Signaling Technology, catalog no. 3900; 1:50) for 1.5 hours at room temperature under gentle rotation. Thereafter, protein G–coated Dynabeads (Thermo Fisher Scientific, catalog no. 10003D) were added, followed by an additional 30 min of incubation at room temperature. The beads were then washed three times with PBS, and immunoprecipitates were boiled for 10 min in 1× Laemmli sample buffer (Bio-Rad, catalog no. 1610747) and were then subjected to SDS-PAGE and immunoblotting.

### Determination of APPsα, APLP2sα, SCF, and METRNL binding to KIT

HEK-293T cells were seeded in 12-well plates and transfected with 28.5 nM siRNA directed against *KIT* as described above using Opti-MEM and Lipofectamine RNAiMAX according to the manufacturer’s instructions. After 20 to 24 hours, cells were transfected with 0.5 μg per well of eukaryotic expression plasmids carrying cDNAs of wild-type mouse or human KIT or the indicated human KIT mutants (table S4) using Opti-MEM (Gibco, catalog no. 31985062) and Lipofectamine 2000 transfection reagent (Invitrogen, catalog no. 11668019). After 48 hours, cells were used for experiments. Cells were harvested using Cellstripper solution (Corning, catalog no. 25-056-CI). Cells were washed with PBS once and incubated with LIVE/DEAD fixable blue dead cell stain kit (Invitrogen, catalog no. C34961), human APPsα or mouse APLP2sα, human or mouse SCF, and human METRNL at the indicated concentrations for 1 hour at 4°C in PBS buffer. After washing with PBS, cells were fixed with 1% paraformaldehyde (PFA) for 10 min. Cells were again washed with PBS and thereafter incubated with the PE anti-6X His tag antibody [Abcam, catalog no. ab237338; 1:100 in PBS containing 1% bovine serum albumin (BSA)], peridinin-chlorophyll-protein complex (PerCP) streptavidin (BD, catalog no. 554064; 1:100 in PBS containing 1% BSA), or Alexa Fluor 488 anti-GST tag antibody (Invitrogen, A-11131; 1:100 in PBS containing 1% BSA) for 30 min at room temperature. After washing with PBS, the samples were analyzed by flow cytometry (BD, FACSMelody). Cells were gated by forward scatter area (FSC-A)/side scatter area (SSC-A), followed by singlet selection using forward scatter height/width (FSC-H/W) and side scatter height/width(SSC-H/W). Live cells were defined by exclusion of dead-cell dye-positive events, and mean fluorescence intensity of phycoerythrin (PE), PerCP, or Alexa Fluor 488 was measured (fig. S10).

### Determination of SCF concentration

Confluent HUVECs in 12-well plates were washed with PBS, and basal medium (EBM-2; Lonza, catalog no. CC-3156) containing no serum and supplements was added. After 90 min, medium was collected and centrifuged to remove debris. The SCF Human ELISA Kit (Invitrogen, catalog no. EHKITLG) was used to measure the SCF levels in medium.

### Animal models

All mice were backcrossed onto a C57BL/6 background at least 8 to 10 times, and experiments were performed with littermates as controls. Male and female animals (8 to 20 weeks of age) were used as indicated. Mice were housed under a 12-hours light-dark cycle with free access to food and water and under specific pathogen–free conditions. Mice carrying a floxed allele of *App* or *Aplp2* and Appsα knock-in as well as AppΔCT15 knock-in mice have been described (*27*, *28*). Mice carrying floxed *App* and *Aplp2* alleles were crossed with Cdh5-CreER^T2^ mice (*26*) to obtain animals with inducible endothelium–specific deficiency (Cdh5-CreER^T2^;*App*^fl/fl^;*Aplp2*^fl/fl^, Cdh5-CreER^T2^;*App*^fl/fl^ and Cdh5-CreER^T2^;*Aplp2*^fl/fl^). For RiboTag analysis, Cdh5-CreER^T2^ mice were crossed with *Rpl22*^tm1.1Psam^ animals (the Jackson Laboratory, stock no. 029977). Cre-mediated recombination was induced by intraperitoneal injection of 1 mg tamoxifen dissolved in Miglyol 812 on 5 consecutive days. Mice carrying floxed alleles but no Cre transgene and also treated with tamoxifen were used as controls. Animal numbers used for each experiment are provided in the figure legends.

### Isolation of cardiac endothelial cells

Mice were euthanized, and hearts were perfused with ice-cold PBS. The hearts were harvested and dissected into small pieces in Hanks’ balanced salt solution (HBSS). Heart pieces were then transferred to gentleMACS C tube (Miltenyi Biotec, catalog no. 130-093-237) with enzymatic digestion buffer containing collagenase II (2 mg/ml; Worthington, catalog no. LS004176) and dispase (1.2 U/ml; Thermo Fisher Scientific, catalog no. 17105041) in DMEM and placed inside gentleMACS Dissociator, where it underwent protocol C (preprogrammed by manufacture). DMEM (5 ml) with 10% FBS was added to samples to stop digestion. After filtration using 100- and 40-μm cell strainers, samples were washed twice with DMEM containing 10% FBS. Debris in the cell suspension was removed using Debris Removal Solution (Miltenyi Biotec, catalog no. 130-10-398), and samples were then incubated with mouse CD31 [platelet endothelial cell adhesion molecule 1 (PECAM1)] microbeads (Miltenyi Biotec, catalog no. 130-097-418) in PBS containing 0.5% BSA and 2 mM EDTA. CD31 (PECAM1)–positive cells were enriched by magnetic-activated cell sorting (MACS) using the AutoMACS system and were then further sorted using a fluorescence-activated cell sorter (BD, FACSMelody) after staining with PE-Cy7 anti-CD31 (PECAM1) antibody (eBioscience, catalog no. 25031181; 1:100). Cells were then centrifuged at 300*g* for 5 min to obtain a cell pellet for further use.

### Expression analysis

Hearts were collected at the indicated time after MI and were dissected under a microscope to separate the infarct, border, and remote zones. The border zone was defined as the region 400 μm around the pale infarct zone (*77*). Endothelial cells were isolated as described above, and total RNA was isolated from snap-frozen tissues and isolated cells using the Quick-RNA MicroPrep Kit (Zymo Research). Quality control of RNAs was done using a NanoDrop ND-100 Spectrophotometer. RNA was reverse transcribed using the ProtoScript II First Strand cDNA Synthesis Kit (New England Biolabs) according to the manufacturer’s instructions. Quantitative real-time PCR was performed using SYBR green PCR mix (Applied Biosystems, catalog no. 4368708) using a Light Cycler 480 II (Roche) and QuantStudio 1 (Applied Biosystems). Gene expression was normalized to the endogenous control (*Gapdh/GAPDH* or *Rn18s/RNA18S1*) and calculated using the ∆∆Ct method. If not otherwise indicated, the average of the respective control/basal condition is set to 1. Primer sequences used for quantitative reverse transcription (qRT)–PCR are shown in table S5.

### RiboTag analysis

Immunoprecipitation and purification of ribosome-associated RNA was performed as described previously (*78*) with modifications. Briefly, hearts from induced Cdh5-CreER^T2^;*Rpl22*^tm1.1Psam^ mice (EC-RiboTag) were perfused through the right ventricle with cold HBSS containing cycloheximide (100 μg/ml; Sigma-Aldrich, catalog no. 01810) and dissected before snap freezing in liquid nitrogen. Tissues were homogenized in polysome buffer [50 mM tris-HCl (pH 7.4), 100 mM KCl, 12 mM MgCl_2_, cOmplete ULTRA (Roche), 1% NP-40, 1 mM dithiothreitol (DTT), SUPERasein (200 U/ml; Thermo Fisher Scientific, catalog no. AM2696), cycloheximide (100 μg/ml), and heparin (1 mg/ml)] with Pestle (ARgos) and clarified by centrifugation at 20,000*g* for 10 min at 4°C. Before immunoprecipitation, 5% of supernatants were kept as input, and the remaining supernatants were mixed with anti–HA-tag Magnetic Beads (Medical & Biological Laboratories, catalog no. M180-11) and rotated for 8 hours at 4°C. Magnetic beads were washed three times for 5 min in high salt buffer [50 mM tris-HCl (pH 7.4), 300 mM KCl, 12 mM MgCl_2_, 1% NP-40, 1 mM DTT, and cycloheximide (100 μg/ml)]. QIAGEN RLT buffer was added to magnetic beads, and total RNA was isolated by RNeasy Micro Kit (QIAGEN) combined with on-column deoxyribonuclease (DNase) digestion (RNase-Free DNase Set, QIAGEN) to avoid contamination by genomic DNA. RNA and library preparation integrity were verified with LabChip Gx Touch (PerkinElmer). RNA amounts were normalized, and 250 ng of total RNA was used as input for the Watchmaker mRNA Library Prep Kit following the manufacturer’s protocol (Watchmaker Genomics). Sequencing was performed on the NextSeq2000 platform (Illumina) using P3 flow cell with 72–base pair single-end setup.

### RNA sequencing analysis

Trimmomatic version 0.39 was used to trim reads after a quality drop below a mean of Q15 in a window of 5 nucleotides (nt) and keeping only filtered reads longer than 15 nt (*79*). Reads were aligned versus Ensembl mouse genome version mm39 (Ensembl release 109) with STAR 2.7.11a (*80*). Alignments were filtered to remove: duplicates with Picard 3.1.1 [Picard: a set of tools (in Java) for working with next generation sequencing data in the BAM format], multi-mapping, ribosomal, or mitochondrial reads. Gene counts were established with featureCounts 2.0.4 by aggregating reads overlapping exons on the correct strand excluding those overlapping multiple genes (*81*). The raw count matrix was normalized with DESeq2 version 1.36.0 (*82*). Contrasts were created with DESeq2 on the basis of the raw count matrix. The Ensemble annotation was enriched with UniProt data [activities at the Universal Protein Resource (UniProt)].

### Myocardial infarction

MI was induced in 10- to 12-week-old mice by ligation of the left anterior descending (LAD) coronary artery about 2 weeks after tamoxifen injection. Mice were anesthetized with isoflurane followed by tracheal intubation. LAD coronary artery ligation was performed under a microscope using a 8-0 suture (Johnson and Johnson) through a lateral thoracotomy at the third intercostal space. Myocardial ischemia was confirmed by observing color changes in the segment of the left ventricle subjected to coronary flow occlusion. Mice were then treated with buprenorphine (0.1 mg/kg subcutaneous) twice daily and metamizole in drinking water (200 mg/kg = 0.8 ml/500 ml) for 3 days. Surgery was performed by an individual who was blinded to the identity of the mouse genotype. Cardiac function was monitored by echocardiography. Cardiac MRI measurement for each mouse was performed before and 3 weeks after MI, and hearts were collected 3 weeks after surgery for further histology and immunofluorescence staining.

### Echocardiography

Transthoracic echocardiograpy was performed before and after surgery at the indicated time points, using a Vevo2100 ultrasound system equipped with MS550D probe (VisualSonics, Fujifilm). Left ventricular ejection fraction (LVEF) and left ventricular endsystolic and enddiastolic volume (LVESV and LVEDV, respectively) were determined by Simpson’s method. The circumference of the akinetic area and left ventricular FAC were determined at the midventricular short-axis plane at the level of the papillary muscle. Measurements were performed by an individual who was blinded to the identity of the mouse genotype.

### Magnetic resonance imaging

Cardiac MRI measurements are performed on a 7.05T Bruker Pharmascan (Bruker, Ettlingen, Germany), equipped with a gradient system at 760 mT/m, using a cryogenically cooled, four-channel–phased array element 1H receiver-coil (CryoProbe), a 72-mm room temperature volume resonator for transmission, and the IntraGate self-gating tool (*83*). The parameters for identification of the ECG were adapted for one heart slice and transferred afterward to the navigator signals of the remaining slices to guarantee in-phase reconstruction of all pictures. Measurements were based on the gradient echo method (repetition time, 6.2 ms; echo time, 1.3 ms; field of view, 2.20 cm by 2.20 cm; slice thickness, 1.0 mm; matrix, 128 × 128; and oversampling, 100). The imaging plane was localized using scout images showing the two- and four-chamber view of the heart, followed by acquisition in short axis view, orthogonal on the septum in both scouts. Multiple contiguous short-axis slices consisting of 7 to 10 slices were acquired for complete coverage of the left and right ventricle. Mice were measured under volatile isoflurane (1.5 to 2.0% in oxygen and air with a flow rate of 1.0 liter/min) anesthesia; the body temperature was maintained at 37°C by a thermostatically regulated water flow system during the entire imaging protocol. MRI data were analyzed using Medis Suite Qmass digital imaging software (Medis, Leiden, The Netherlands).

### Plasma CKMB measurement

Mouse blood was collected from the tail vein into EDTA tubes (Sarstedt; Microvette 500 K3E) 24 hours after LAD coronary artery ligation. After centrifugation (300*g* for 15 min), the plasma was collected. To determine plasma levels of the creatine kinase myocardial band (CKMB) isoenzyme, a mouse CKMB ELISA Kit (Elabscience, catalog no. E-EL-M0355) was used.

### Analysis of in vivo blood perfusion and endothelial proliferation

To label proliferating cells in the heart after MI, EdU (Lumiprobe GmbH, catalog no. 40540) was administered via the drinking water (0.5 mg/ml) for 3 days before mice were euthanized. In addition, to visualize histologically the perfused vasculature, Alexa Fluor 647 Isolectin GS-B4 (IB4) conjugate (Invitrogen, catalog no. I32450) was injected (12.5 μg per mouse) via the tail vein under isoflurane anesthesia 15 min before the mice were euthanized. Subsequently, perfusion with ice-cold PBS was performed, and the hearts were harvested. After sectioning and staining of infarcted hearts, the area negative for cardiomyocyte markers was defined as the “infarct region,” the area of 400 μm around the infarct zone was defined as the “border zone,” and the rest was regarded as “remote zone” (*77*).

### AAV2 infection

AAV2-QuadYF (*30*, *31*) carrying the cDNA encoding mouse APPsα was generated by VectorBuilder. To generate AAV2-QuadYF viral particles, a construct encoding APPsα and mCherry separated by the self-cleaving T2A linker peptide from thosea asigna virus under the control of the murine Tie1 promoter was used (pAAV[Exp]-Tie1 > {signal peptide-2xHA-mAPPalpha(ns)}: T2A:mCherry, VB230620-1261jna). Mice were briefly anesthetized by isoflurane inhalation, and QuadYF-AAV2-Tie1-Appsα-mCherry or mCherry control QuadYF-AAV2 (1 × 10^12^ viral genomes in 100 μl of PBS) were administered via the tail vein. After 2 weeks, LAD artery ligation to induce MI was performed as described above.

### Analysis of immune cell infiltration in the heart

Immune cell infiltration in the heart was analyzed as described previously (*84*) with modifications. After euthanasia, mice were perfused through the left ventricle with PBS. Hearts were then dissected as described above and placed in cold PBS. Tissues were finely minced with a scalpel and transferred to 3 ml of digestion solution [DMEM (Gibco, catalog no. 10938025) and collagenase I (450 U/ml; Sigma-Aldrich, catalog no. C0130), DNase I (60 U/m; Sigma-Aldrich, catalog no. DN25), and hyaluronidase (60 U/ml; Sigma-Aldrich, catalog no. H3506)] and were incubated at 37°C for 1 hour with gentle agitation. After digestion, the reaction was stopped by addition of 8 ml of ice-cold enzyme deactivation buffer (Thermo Fisher Scientific, catalog no. 14175129), 1% FBS (Thermo Fisher Scientific, catalog no. 10270106), and 0.1% BSA (Sigma-Aldrich, catalog no. A7030). The suspension was filtered through a 40-μm cell strainer, centrifuged for 6 min at 400*g* at 4°C, resuspended in 1 ml of ACK lysis buffer (Thermo Fisher Scientific, catalog no. A1049201), and incubated for 5 min at room temperature. Then, 9 ml of cold DMEM was added to the samples, and they were centrifuged at 400*g* for 6 min at 4°C. The resulting pellet was resuspended in 1 ml of fluorescence-activated cell sorting (FACS) buffer [PBS (pH 7.4), 2% FBS, and 2 mM EDTA] and again centrifuged for 5 min at 400*g* at 4°C. For the T cell panel as well as for the analysis of macrophage and neutrophil activation, the pellet was resuspended in FACS buffer and either stained with LIVE/DEAD fixable blue dead cell stain (Thermo Fisher Scientific, catalog no. L23105) (T cell panel) or was blocked with Fc blocker and stained with the respective antibody cocktail (macrophage and neutrophil activation panels, see table S6) (*85*). For the myeloid cell panel, the pellet was resuspended in 90 μl of autoMACS buffer [PBS (pH 7.4), 0.5% BSA, and 2 mM EDTA] and 10 μl of anti-mouse CD45 microbeads (Miltenyi Biotec, catalog no. 130-052-301) and was incubated for 15 min at 4°C. autoMACS buffer (1 ml) was added to the tubes. After centrifugation at 300*g* for 10 min at 4°C, the pellet was resuspended in 2 ml of autoMACS buffer, and CD45-positive cells were purified using an autoMACS device (Miltenyi Biotec) with the program Possel. The positive fraction was centrifuged at 400*g* for 5 min and each sample was resuspended in 90 μl of FACS buffer. Fc receptors were blocked by incubation with 2 μl of anti-mouse Cd16/Cd32 Fc blocker (eBioscience, catalog no. 14-0161-85) for 15 min at 4°C. Then, an antibody cocktail shown in table S6 was added, and samples were incubated at 4°C for 40 min. After washing twice with cold FACS buffer, the samples were resuspended in FACS buffer with DAPI (1 μg/ml; Invitrogen, catalog no. D3571) and counting beads (Spherotech, catalog no. ACFP-100-3) and analyzed on a FACSCanto II using BD Diva software. Data were analyzed with FlowJo software. For the T cell panel, after LIVE/DEAD cell staining for 30 min on ice, samples were washed two times with FACS buffer and resuspended in 38 μl of FACS buffer with 2 μl of anti-mouse Cd16/Cd32 Fc blocker and 50 μl of BD Horizon Brilliant Stain Buffer (BD, catalog no. 563794). After 15 min of incubation on ice, 5 μl of TrueStain Monocyte blocker (BioLegend, catalog no. 426102) was added to the samples before adding the specific antibody cocktail (table S6) for analyzing T cells and further incubation for 40 min at 4°C. After two washes, samples were resuspended in 100 μl of FoxP3 fixation buffer (from FoxP3/Transcription Factor Staining Buffer Set; eBioscience, catalog no. 00-5523-00) and incubated on ice for 30 min. After centrifugation, samples were resuspended and stained for 30 min on ice with 100 μl of intracellular antibody staining mix (2 μl of anti-CD152 and 2 μl of anti-FoxP3 antibodies) in 96 μl of 1× permeabilization buffer from the FoxP3/Transcription Factor Staining Buffer Set (eBioscience, catalog no. 00-5523-00). After one wash with 1× permeabilization buffer, the samples were resuspended in FACS buffer with counting beads and analyzed on a BD LSR Fortessa flow cytometer (*86*).

### Histology and immunostaining

After euthanizing the mice, hearts were perfused with cold PBS and fixed in PBS containing 4% PFA. After overnight incubation at 4°C, hearts were consecutively incubated in PBS containing increasing concentrations of sucrose (10, 20, and 30%; Sigma-Aldrich, catalog no. S0389) overnight at 4°C. Tissues were then cryopreserved in OCT compound (Tissue-Tek) and were cryosectioned at a thickness of 8 to 10 μm. For immunofluorescence staining, cryosections were incubated in PBS containing 0.3% Triton X-100 twice for 5 min to permeabilize the tissue and were blocked in PBS containing 5% normal goat serum for 30 min at room temperature. Sections were incubated overnight at 4°C with primary antibodies diluted in blocking solution and were then washed three times for 5 min in PBS and incubated with the appropriate Alexa Fluor 405– or Alexa Fluor 488–conjugated secondary antibodies for 1 hour at room temperature (if the primary antibody was not conjugated). Nuclei were stained with DAPI (1 ng/ml; Invitrogen, catalog no. D3571). After being washed with PBS, slices were mounted in Aqua-polymount (Polyscience) and were imaged using confocal laser microscopy (Leica SP5). Quantification was performed by taking five to seven pictures from each slide followed by analysis with ImageJ (Fiji, version 2.14.0). Antibodies used for immunohistochemistry are shown in table S7.

Heart tissues of mice that had received EdU were processed for EdU staining with Click-iT EdU Alexa Fluor 555 kit (Invitrogen, catalog no. 10338) according to the manufacturer’s instructions. After EdU staining, costaining was continued as described above. For scar size measurement, heart tissues were embedded in paraffin after overnight fixation with 4% PFA. Scar tissue was delineated by Masson’s trichrome (Sigma-Aldrich, catalog no. HT15) staining according to the manufacturer’s instruction.

### Preparation of MLECs

To isolate MLECs, lungs were harvested and dissected into small pieces in ice-cold PBS, and lung pieces were incubated in gentleMACS C tubes (Miltenyi Biotec, catalog no. 130-093-237) in 5 ml of lung digestion buffer containing collagenase II (1 mg/ml; Worthington, catalog no. LS004176), collagenase IV (2.5 mg/ml; Worthington, catalog no. LS004189), and DNase I (15 μg/ml; Sigma-Aldrich, catalog no. D4527) for 30 min at 37°C under gentle agitation. The reaction was halted with 5 ml of DMEM containing 10% (v/v) FBS. The digest was then sequentially filtered through 100- and 40-μm cell strainers and centrifuged at 300*g* for 5 min, and the pellet was resuspended in 5 ml of Ammonium-Chloride-Potassium (ACK) lysis buffer on ice for 3 min to remove red blood cells. Cells were washed twice by centrifugation at 300*g* for 5 min and were resuspended in 2 ml of PBS containing 0.5% BSA and 2 mM EDTA and were incubated with mouse anti-CD31 microbeads (Miltenyi Biotec, catalog no. 130-097-418). After incubation for 15 min at 4°C, CD31-positive cells were isolated by MACS using the AutoMACS system and were washed again as described above.

### Prediction of protein complex by AlphaFold

AlphaFold2 was used to predict protein-protein interactions. The amino acid sequences of the target proteins were obtained from the UniProt database and input to the AlphaFold2 run in ChimeraX [the Resource for Biocomputing, Visualization, and Informatics at the University of California, San Francisco, with support from National Institutes of Health (R01-GM129325) and the Office of Cyber Infrastructure and Computational Biology, National Institute of Allergy and Infectious Diseases] (*87*), and the iPTM score was analyzed.

### Evaluation of RNA sequencing data

To analyze the expression of genes encoding transmembrane proteins including *App* and *Aplp2* in single endothelial cells of hearts of healthy mice or after MI, we used data from Tombor *et al.* (*88*). To analyze expression of *APP* and *APLP2* in human hearts, we used public data from Kanemaru *et al.* (*89*). To analyze expression of *APP*, *APLP2*, *ADAM9*, *ADAM10*, *ADAM17*, *KIT*, and *KITL* in single cardiac endothelial cells of human patients with acute MI and healthy control individuals, we used data from Kuppe *et al.* (*90*). All data were downloaded as preprocessed (if provided) or raw data from the respective repositories and further prepared for visualization and exploration in cellxgene (v1.1.1) via the sc-framework workflows (https://doi.org/10.5281/zenodo.14524557). Graphs were generated either by scanpy (https://genomebiology.biomedcentral.com/articles/10.1186/s13059-017-1382-0; v1.9.8) or by the cellxgene VIP plugin (https://github.com/interactivereport/cellxgene_VIP). To analyze expression of genes encoding transmembrane proteins including APP and APLP2 in HCMVECs, we used preanalyzed data from Bao *et al.* (*91*).

### Statistics

Statistical analysis was performed using the GraphPad Prism software (9.3.1) from GraphPad Software Inc. (La Jolla, CA, USA). The data are presented as means ± SEM, and “*n*” represents the number of independent experiments. Data were tested for normality using the Shapiro-Wilk test. Statistical analysis between two groups was performed using unpaired or paired two-tailed Student’s *t* test, while multiple group comparisons were analyzed with one-way analysis of variance (ANOVA) followed by Tukey’s or Šidák’s post hoc test. For datasets that did not follow normal distribution, Mann-Whitney test was used for the comparison of two groups, and Kruskal-Wallis test followed by Dunn’s multiple comparison was performed for multiple group comparisons. Comparisons between multiple groups at different time points were performed using two-way repeated-measures ANOVA with post hoc multiple comparison tests. In survival analysis, Kaplan-Meyer estimator was performed for survival curves, followed by the Gehan-Breslow-Wilcoxon test. A *P* value of less than 0.05 was considered to be statistically significant.

### Study approval

All procedures involving animal care and use in this study were approved by the local animal ethics committees [Regierungspräsidium Darmstadt, Germany (B2/2005) and Nagoya University School of Medicine, Japan (M250121)].
