# Supplementary material for: Endothelial soluble APP/APLP2 promote heart repair through KIT-mediated angiogenesis
Source: Sci Adv. 2026 May 22;12(21):eaeh0301. doi: 10.1126/sciadv.aeh0301 (PMC13196746; doi:10.1126/sciadv.aeh0301)
Supplement: Supplementary file 1 — Figs. S1 to S10 Tables S1 to S7 [file sciadv.aeh0301_sm.pdf]

Science Advances
AAAS

Supplementary Materials for

Endothelial soluble APP/APLP2 promote heart repair through KIT-mediated angiogenesis

Haruya Kawase *et al.*

Corresponding author: Stefan Offermanns, stefan.offermanns@mpi-bn.mpg.de

Sci. Adv. **12**, eaeh0301 (2026)
DOI: 10.1126/sciadv.aeh0301

This PDF file includes:

Figs. S1 to S10
Tables S1 to S7

Supplementary Figures

Supplementary Fig. 1

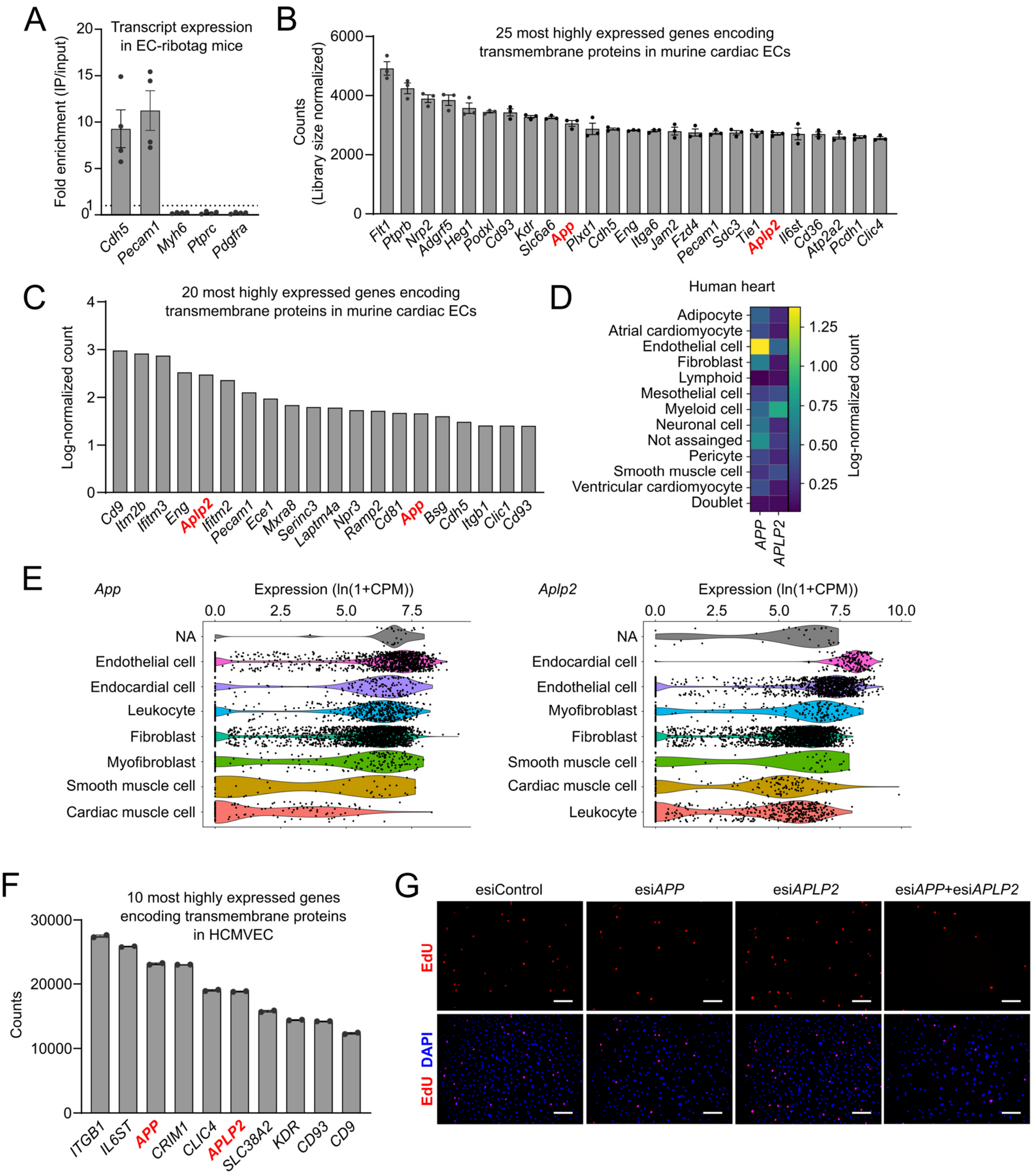

Suppl. Figure 1. Expression of genes encoding APP and APLP2 in cardiac endothelial cells. A, Enrichment of endothelial cell RNAs using the EC-RiboTag mouse. Cdh5-CreER^{T2};*Rpl22*$^{tm1.1Psam}$ mice (EC-RiboTag) were euthanized, hearts were removed and homogenized, and ribosomes of endothelial cells containing HA-tagged RPL22 protein were immunoprecipitated. qRT-PCR analysis was performed with the homogenates (input) and with the immunoprecitpitates using cell type-specific markers (*Cdh5* and *Pecam1*

(endothelial cells), *Myh6* (cardiomyocytes), *Ptprc* (leukocytes) and *Pdgfra* (fibroblasts)). Shown is the ratio of the signal normalized to the *Rn18s* expression obtained in the immunoprecipitates and in the input material (n=4 independent experiments). **B**, Ribosome-associated RNA in cardiac endothelial cells was purified from EC-RiboTag mice, and RNA-seq was performed (n=3). Of the about 3000 detected genes which encode plasma membrane proteins, the 25 most highly expressed genes, including *App* and *Aplp2*, are shown. **C**, RNAseq-derived gene expression in single endothelial cells from murine hearts (Tombor et al., 2021) was analyzed (n=551 single cells). The 20 most highly expressed genes encoding transmembrane proteins including *App* and *Aplp2*, are shown. **D**, Publicly available single-cell RNA-seq data of human hearts (Heart Cell Atlas) (Kanemaru et al., 2023) were analyzed, and expression levels of *APP* and *APLP2* in different cardiac cell types is shown in the heatmap. **E**, Publicly available single-cell RNA-seq data of mouse hearts (Tabula Muris Consortium, 2018). Violin plots show the expression levels of *App* and *Aplp2* in different murine cardiac cell types. **F**, Publicly available RNA-seq data of human primary cardiac microvascular endothelial cells (HCMVECs, GSE93705) (Bao et al., 2017) were analyzed, and the 10 most highly expressed genes encoding transmembrane proteins, including *APP* and *APLP2*, are shown. **G**, Proliferation of HUVECs was analyzed by determining EdU incorporation. 24 hours after transfection with control esiRNA or esiRNA against *APP* and/or *APLP2*, HUVECs were incubated with EdU (10 μM) for 12 hours and the percentage of proliferating EdU-positive cells was determined (n=12 different wells). Shown are representative images counterstained with DAPI of the statistical analysis shown in Fig. 1E; bar length: 200 μm. Shown are mean values ± S.E.M..

Supplementary Fig. 2

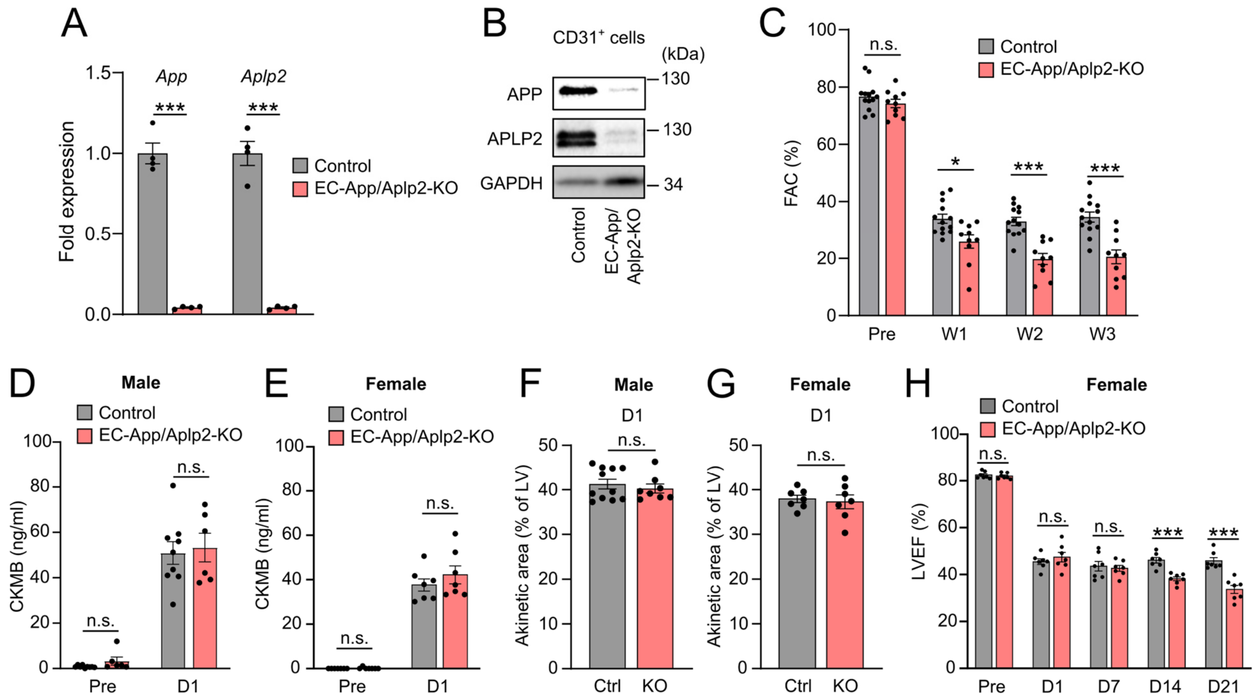

Suppl. Figure 2. Knock-out efficiency and initial infarct extent in EC-App/Aplp2-KO mice. A, **B**, CD31+ cells were isolated by magnetic-activated cell sorting from the hearts of control and EC-App/Aplp2-KO mice (n=4 mice per group) 2 weeks after tamoxifen injection, and expression of *App* and *Aplp2* was analyzed by qRT-PCR (A) or immunoblotting (B). **C**, The fractional area change (FAC) was determined before (Pre) and 1-3 weeks after MI in control (n=13) and EC-App/ALP2-KO mice (n=10). **D**, **E**, Plasma creatine kinase-MB (CKMB) levels of male (D) and female (E) control (n=9 males and 7 females) and EC-App/Aplp2-KO mice (n=6 males and 7 females) before and day 1 after MI were determined by ELISA. **F**, **G**, Akinetic area of left ventricle (LV) of male (F) and female (G) control (n=11 males and 7 females) and EC-App/Aplp2-KO mice (n=8 males and 7 females) 1 day after MI were assessed by echocardiography. **H**, Left ventricular ejection fraction (LVEF) determined before (Pre) and 1-3 weeks after MI in female control and EC-App/Aplp2-KO mice (n=7 mice per group). Shown are mean values ± S.E.M.; *, $P \leq 0.05$; ***, $P \leq 0.001$; n.s., non-significant (unpaired two-tailed t test (A, F, G), two-way repeated measures ANOVA with Sidak's multiple comparisons test (C-E, H)).

Supplementary Fig. 3

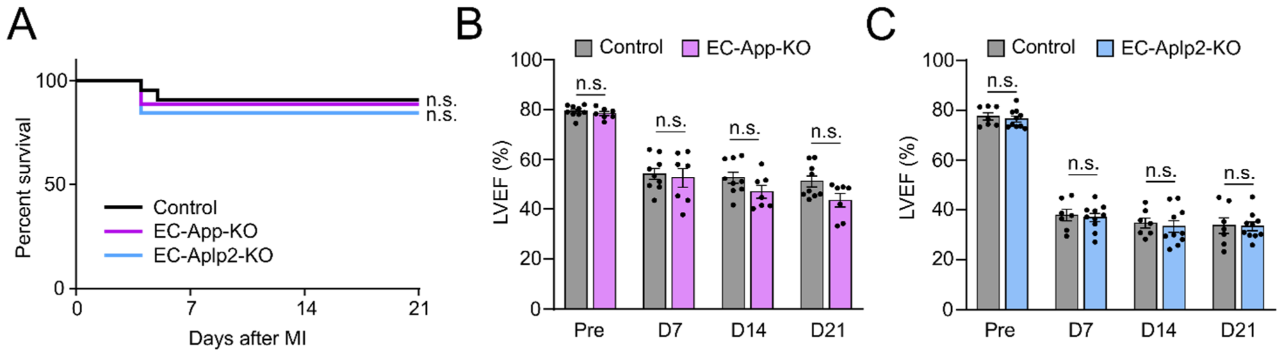

Suppl. Figure 3. Normal response of EC-App-KO and EC-Aplp2-KO mice to MI. A, Survival of control (n=22), EC-App-KO (n=9), and EC-Aplp2-KO mice (n=13) after MI. **B**, **C**, Echocardiographic analysis of left ventricular ejection fraction (LVEF) before and at the indicated time periods after MI in control (n=9) and EC-App-KO (n=7) (B), as well as control (n=7) and EC-Aplp2-KO mice (n=10) (C). Shown are mean values ± S.E.M.; n.s., non-significant (Gehan-Breslow-Wilcoxon test (A), and two-way repeated measures ANOVA with Sidak's multiple comparisons test (B, C)).

Supplementary Fig. 4

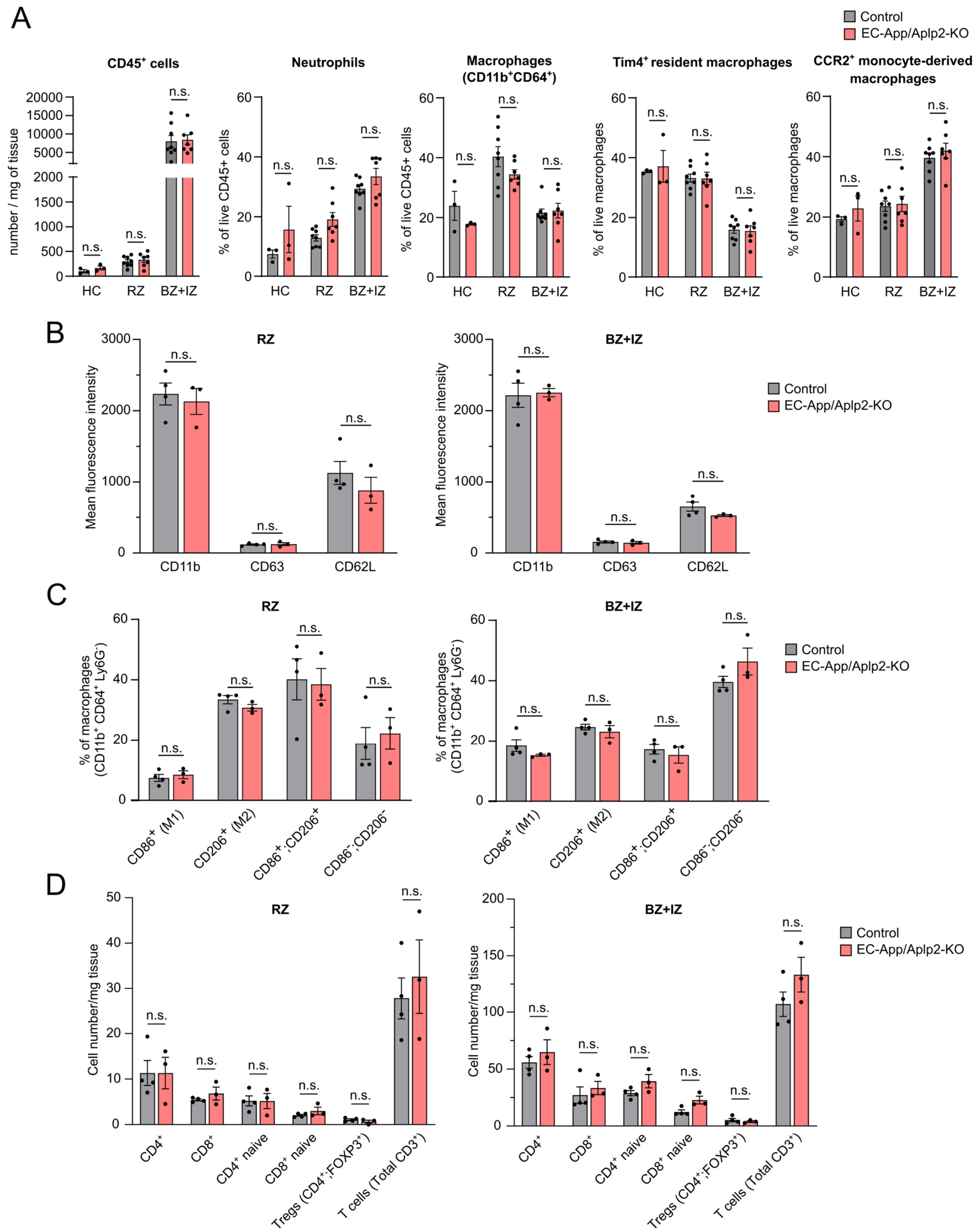

Suppl. Figure 4. Effect of endothelial loss of APP and APLP2 on cardiac immune cells after MI. A-D, Hearts from control and EC-App/Aplp2-KO mice were explanted 3 days after MI, and the remote zone (RZ) as well as the infarct and border zones (IZ and BZ) were prepared. A, different immune cell populations were analyzed by flow cytometry (n=8 mice, control; n = 7 mice, EC-App/Aplp2-KO). Also healthy control (HC) mice were

analyzed (n=3 mice per group). B-D, different immune cell populations were analyzed by flow cytometry (controls (n=4) and EC-App/Aplp2-KO mice (n=3)). Shown are quantifications of different markers reporting neutrophil activation (B), the proportions of M1 and M2 activated macrophages (C) and different T-lymphocyte populations (D). Shown are mean values ± S.E.M.; n.s., non-significant (unpaired t test corrected for multiple testing by two-stage step-up method Benjamini, Krieger and Yekutieli (A); multiple t tests (B-D)).

Supplementary Fig. 5

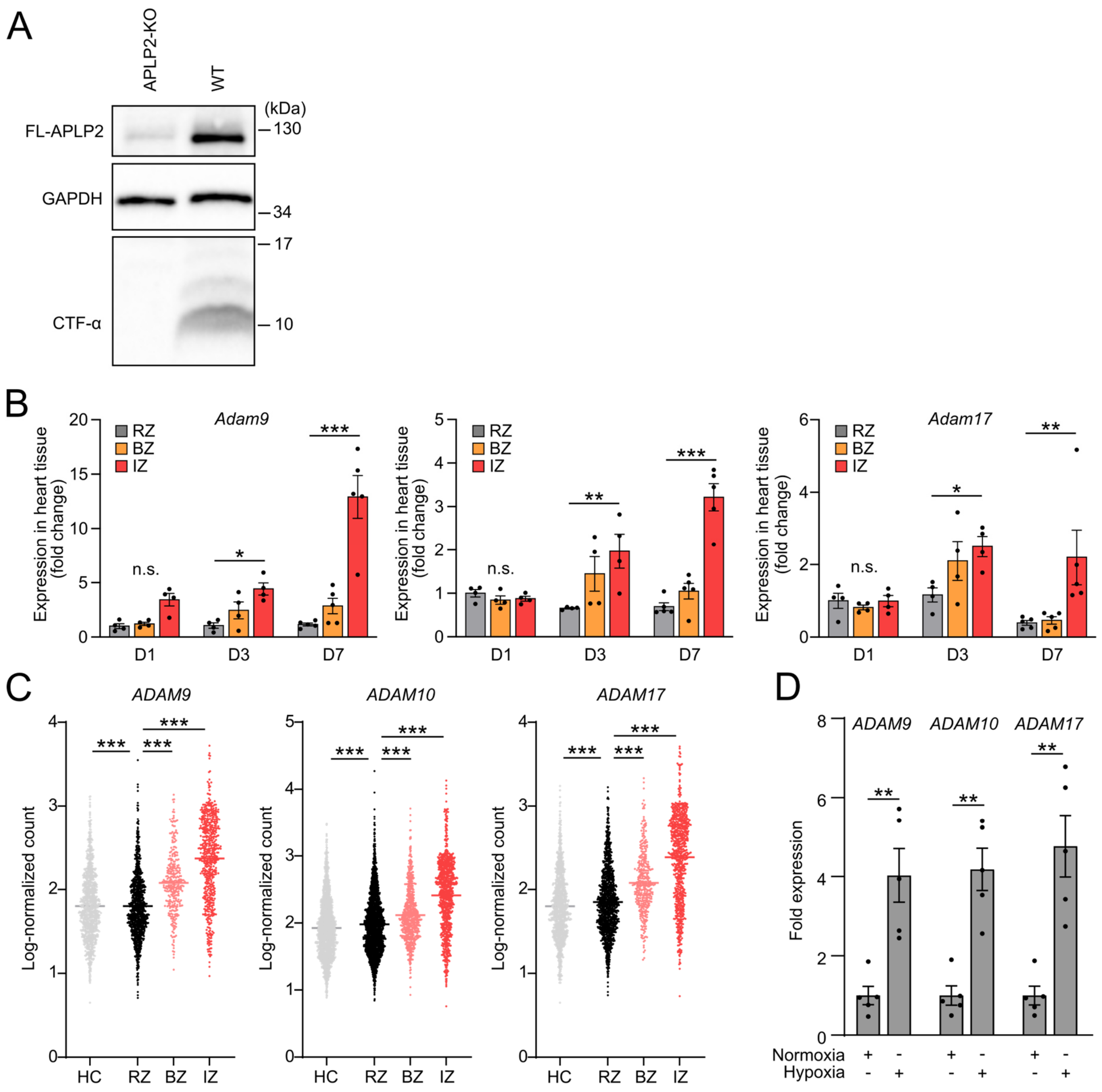

Suppl. Figure 5. Anti-APLP2 antibody specificity and expression of human α-secretases. A, To test the specificity of the anti-APLP2 antibody, cardiac endothelial cells were isolated from wild-type mice (WT) or from Aplp2$^{-/-}$ animals (APLP2-KO), lysed and analyzed by immunoblotting using the anti-APLP2 antibody. **B**, Hearts were collected from wild-type mice at the indicated time points after MI, and the remote zone (RZ), border zone (BZ) and infarct zone (IZ) were prepared. Expression of *Adam9*, *Adam10* and *Adam17* was examined by qRT-PCR (n=4 mice (D1, D3); n=5 mice (D5)). Data were normalized to *Gapdh*, and expression in the RZ at D1 was set as "1". **C**, RNAseq-derived expression levels of *ADAM9*, *ADAM10*, and *ADAM17* in single cardiac endothelial cells from the indicated areas of the heart of patients with acute MI (RZ, remote zone; BZ, border zone; IZ, infarct zone) as well as healthy control individuals (HC) (Kuppe et al., 2022) (each dot represents a single cell; n=6 patients and n=4 healthy controls). **D**, Effect of 8 hours of

hypoxia (1% O_2) on the expression of the indicated α-secretase encoding genes in human cardiac microvascular endothelial cells (HCMVECs) compared to normoxic conditions (n=5 per group). Shown are mean values ± S.E.M.; *, $P \leq 0.05$; **, $P \leq 0.01$; ***, $P \leq 0.001$; n.s., non-significant (two-way ANOVA with Sidak's multiple comparisons test (B), one-way ANOVA with Tukey's multiple comparisons test (C), Mann Whitney test (D)).

Supplementary Fig. 6

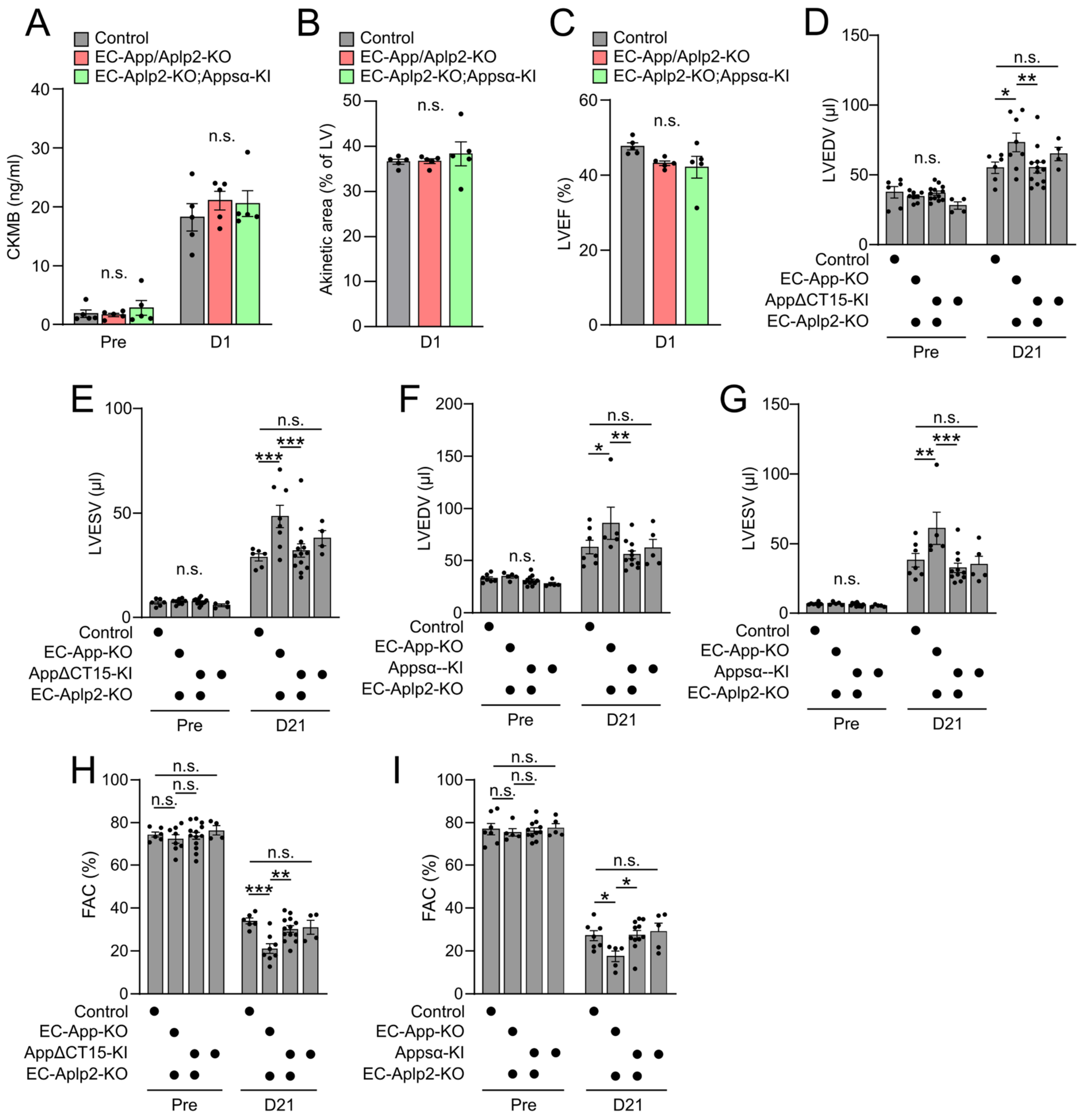

Suppl. Figure 6. Phenotype of APPsα-KI and APPΔCT15-KI mice after MI. A, Plasma creatine kinase-MB (CKMB) levels of control, EC-App/Aplp2-KO, and EC-Aplp2-KO;Appsα-KI mice before and 1 day after MI were determined by ELISA (n=5 mice per group). **B**, **C**, Akinetic area of left ventricle (LV) (B) and left ventricular ejection fraction (LVEF) (C) of control, EC-App/Aplp2-KO, and EC-Aplp2-KO;Appsα-KI mice 1 day after MI were assessed by echocardiography (n=5 mice per group). **D-I**, MI was induced in control (n=3 males, 3 females), EC-App/Aplp2-KO (n= 4 males, 4 females), AppΔCT15-KI (n= 6 males, 7 females) and EC-Aplp2-KO;AppΔCT15-KI mice (n= 1 males, 3 females) (D, E, H) or in control (n= 5 males, 2 females), EC-App/Aplp2-KO (n= 4 males, 1 females), Appsα-KI (n= 3 males, 8 females) and EC-Aplp2-KO;Appsα-KI mice (n= 3 males, 2 females) (F, G, I). Cardiac function was examined by echocardiography. LVEDV, left ventricular end-

diastolic volume (D, F); LVESV, left ventricular end-systolic volume (E, G); FAC, fractional area change (H, I). Shown are mean values ± S.E.M.; *, $P \leq 0.05$; **, $P \leq 0.01$; ***, $P \leq 0.001$; n.s., non-significant (one-way ANOVA with Tukey's multiple comparisons test (B, C), two-way ANOVA with Tukey's multiple comparisons test (A, D-I)).

Supplementary Fig. 7

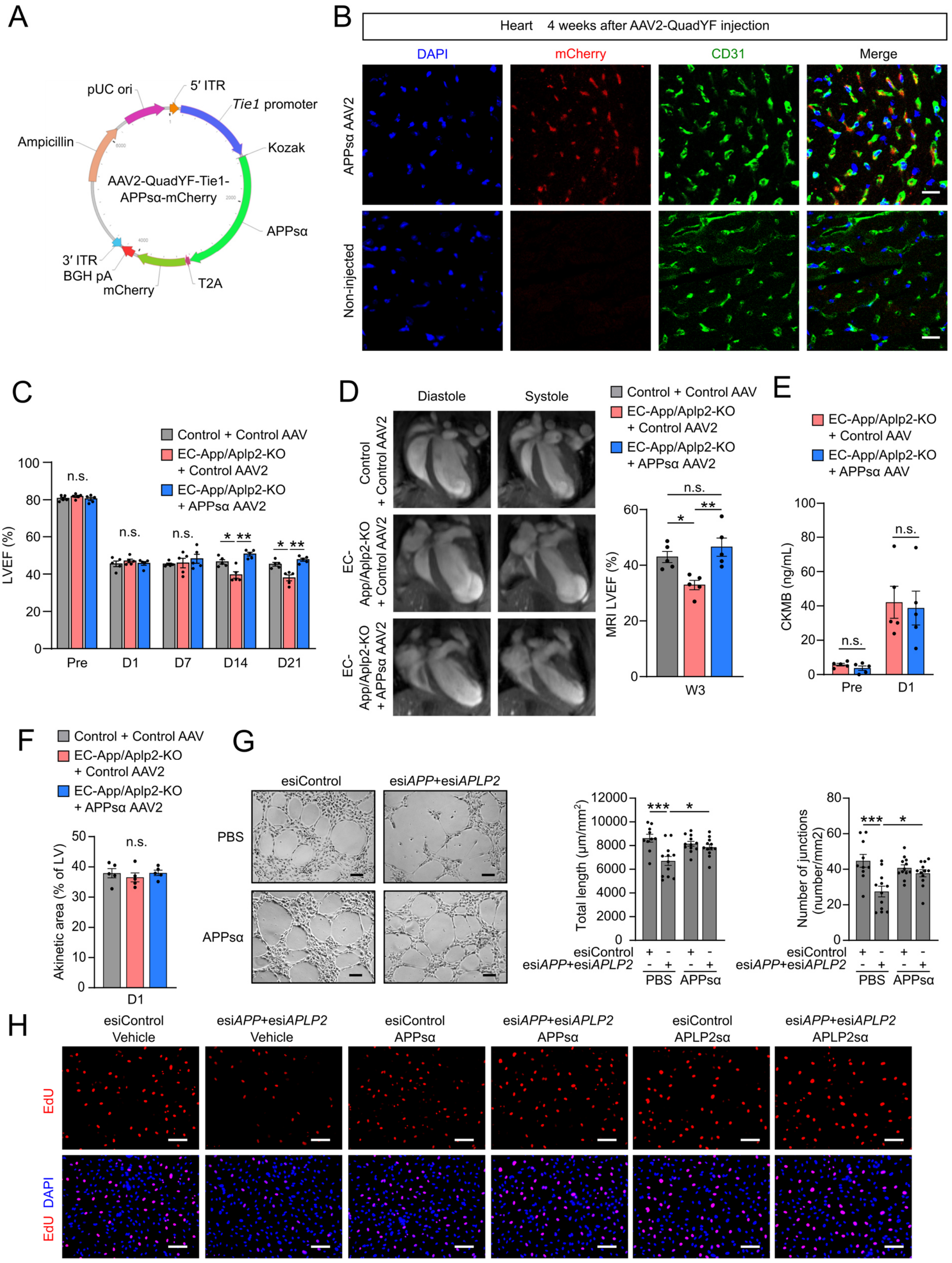

Suppl. Figure 7. AAV2-mediated endothelial expression of APPsα. A, Map of vector used to generate AAV2 particles transducing APPsα and mCherry separated by the self-cleaving T2A peptide under the control of the *Tie1* promoter. The construct was used to generate quadruplet mutant AAV2 viral particles referred to as AAV2-QuadYF. **B**, Images

of heart sections from non-infected mice and mice infected with AAV2-QuadYF transducing APPsα and mCherry stained with DAPI and with antibodies against mCherry and CD31 (shown is a representative of 3 independently performed experiments). Scale bar: 20 µm. **C**, EC-App/Aplp2-KO mice were infected with AAV2-QuadYF transducing APPsα and mCherry (APPsα AAV2) or only mCherry (control AAV2) by i.v. injection 2 weeks before MI. Left ventricular ejection fraction (LVEF) was examined by echocardiography before (Pre) and at the indicated time periods after induction of MI (n=5 mice per group). **D**, Cardiac function was analyzed by magnetic resonance imaging 3 weeks after MI in EC-App/Aplp2-KO mice infected with control AAV2 or AAV2 transducing APPsα (n=5 mice per group). Representative images at diastole and systole are shown. The bar diagram shows the statistical evaluation of the left ventricular ejection fraction (LVEF). **E**, Plasma creatine kinase-MB (CKMB) levels of EC-App/Aplp2-KO mice infected with control AAV2 or AAV2 transducing APPsα (n=5 mice per group) before (Pre) and 1 day after (D1) MI were determined by ELISA (n=5 mice per group). **F**, Akinetic area of left ventricle (LV) of EC-App/Aplp2-KO mice infected with control AAV2 or AAV2 transducing APPsα (n=5 mice per group) 1 day after MI were assessed by echocardiography. **G**, HUVECs transfected with control esiRNA or esiRNAs against *APP* and *APLP2* were seeded in matrigel with or without APPsα to analyze tube formation (n=10 (control, PBS) or n=12 (all other conditions)). The bar diagram shows the statistical evaluation of total tube length and number of junctions. Scale bar: 200 µm. **H,** Proliferation of HUVECs in the absence or presence of APPsα or APLP2sα was analyzed by determining EdU incorporation. 24 hours after transfection with control esiRNA or esiRNA against *APP* and *APLP2*, HUVECs were incubated with EdU (10 µM) in the absence or presence of APPsα (20 nM) or APLP2sα (20 nM), and the percentage of proliferating EdU-positive cells was determined after 12 h (n=8-10 different wells). Shown are representative images counterstained with DAPI of the statistical analysis shown in Fig. 4G; bar length: 100 μm. Shown are mean values ± S.E.M.; *, $P \leq 0.05$; **, $P \leq 0.01$; ***, $P \leq 0.001$; n.s., non-significant (two-way repeated measures ANOVA with Sidak's multiple comparisons test (C, E), one-way ANOVA with Sidak's multiple comparisons test (D, G), Kruskal-Wallis test (F)).

Supplementary Fig. 8

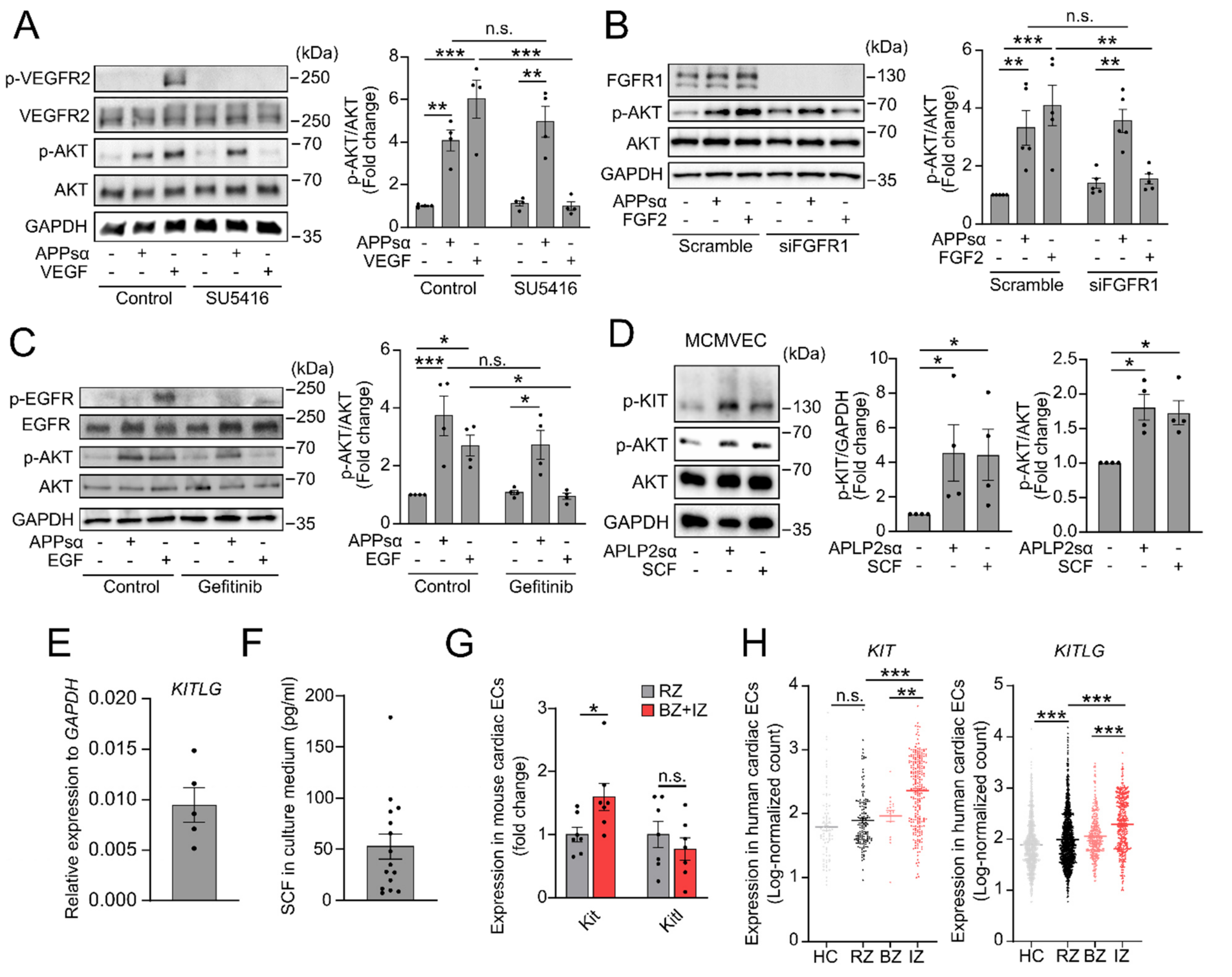

Suppl. Figure 8. APPsα endothelial effects and expression of SCF. A, HUVECs were treated with APPsα (20 nM) or VEGF165a (50 ng/ml) for 10 minutes in the absence or presence of the VEGFR inhibitor SU5416, and phosphorylation of VEGFR2 and AKT was examined by immunoblotting. **B**, HUVECs transfected with control siRNA or siRNA directed against *FGFR1* were treated with APPsα (20 nM) or FGF2 (20 ng/ml) for 10 min, and phosphorylation of AKT was examined by immunoblotting. **C,** HUVECs were treated with APPsα (20 nM) or EGF2 (20 ng/ml) for 10 min in absence or presence of the EGFR inhibitor Gefetinib (10 μM), and the effect on phosphorylation of EGFR and AKT was examined by immunoblotting. Shown are representative of 4-5 independently performed experiments and the statistical analysis (n=4-5) (A-C). **D**, Murine cardiac microvascular endothelial cells (MCMVECs) were treated with mouse APLP2sα (20 nM) or mouse SCF (5 nM) for 10 min, and the effect on KIT and AKT phosphorylation was examined by immunoblotting. Shown is a representative of 4 independently performed experiments and the statistical analysis. **E**, RNA was isolated from cultured human cardiac microvascular

endothelial cells (HCMVECs), and expression levels of *KITLG* were examined by qRT-PCR. Data are shown as ratio of GAPDH (n=5). **F**, SCF levels in the basal culture medium of HUVECs were determined by ELISA (n=15). **G**, Ribosome-associated RNA from endothelial cells was purified from the remote zone (RZ) and border as well as infarct zones (BZ and IZ) of hearts of EC-RiboTag mice 7 days after MI (n=7). Expression levels of *Kit* and *Kitlg* were examined by qRT-PCR. **H**, RNAseq-derived expression levels of *KIT* and *KITLG* in single cardiac endothelial cells from the indicated areas of the heart of patients with acute MI (RZ, remote zone; BZ, border zone; IZ, infarct zone) as well as of healthy individuals (HC). Shown are mean values ± S.E.M.; *, $P \leq 0.05$; **, $P \leq 0.01$; ***, $P \leq 0.001$; n.s., non-significant (one-way ANOVA with Sidak's (A-C) or Tukey's (H) multiple comparisons test, Kruskal-Wallis test with Dunn's multiple comparisons test (D) and paired t-test (G).

Supplementary Fig. 9

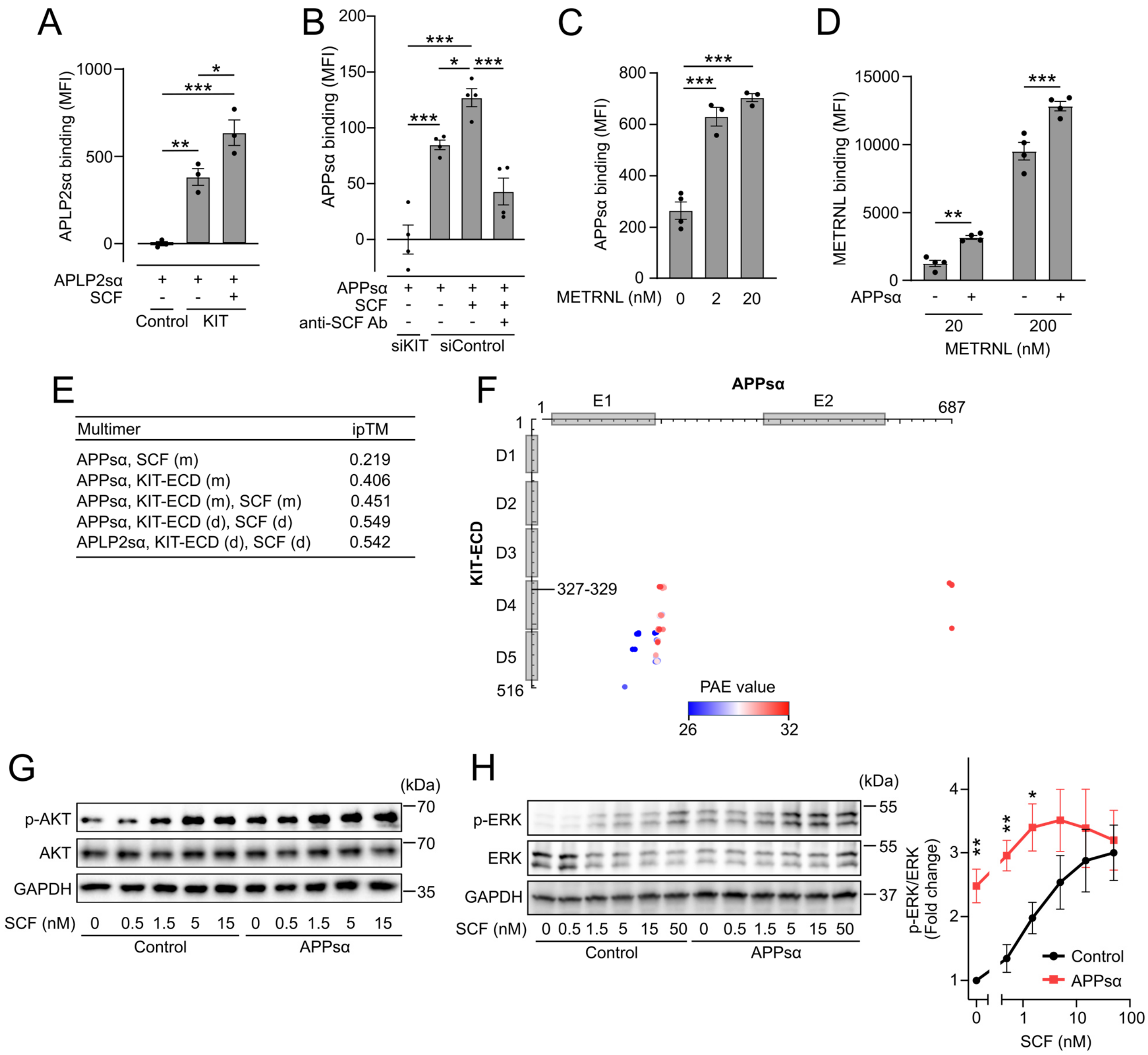

Suppl. Figure 9. Binding of APLP2sα and E1 domain to KIT and AlphaFold-predicted multimers. A, Binding of His-tagged mouse APLP2sα to control HEK-293T cells and cells expressing mouse *Kit* in the absence or presence of 5 nM SCF. Binding was determined by flow-cytometry, and shown is the quantification of the mean fluorescence intensity (MFI) of the anti-His signal (n=3 independent experiments). **B**, Binding of His-tagged APPsα to KIT in HUVECs transfected with siRNA against *KIT* (siKIT) or control siRNA (siControl) in the absence or presence of SCF (5 nM) and a SCF-neutralizing antibody (anti-SCF Ab, 1.0 µg/ml). Binding was determined by flow cytometry, and shown is the quantification of the mean fluorescence intensity (MFI) of the His signal (n=4). **C**, Binding of His-tagged human APPsα to human KIT expressing HEK-293T cells in the absence or presence of the indicated concentrations of human METRNL was determined by flow cytometry after staining of cells with an anti-His antibody. Shown is the mean fluorescence intensity (MFI)

of the anti-His signal (n=3-4). **D**, Effect of human APPsα (300 nM) on binding of GST-tagged human METRNL to KIT-expressing HEK-293 cells as determined by flow-cytometry after staining with anti-GST antibodies. Shown is the mean fluorescence intensity of the anti-GST signal (n=4). **E**, AlphaFold2 predicted interface predicted template modelling (ipTM) scores for the indicated human multimers (KIT-ECD, extracellular part of KIT; m, monomer; d, dimer). **F**, Shown are the sequences of the KIT extracellular domain (KIT-ECD) including domains D1 to D5 and of APPsα including E1 and E2 domains with the positions of predicted interacting amino acid pairs. Each dot represents an amino acid pair with the colour-coded predicted aligned error (PAE). PAE is a confidence measure for the relative position of any two residues within the predicted structure. **G, H**, HUVECs were treated with increasing concentrations of SCF in the absence and presence of 50 nM APPsα, and AKT phosphorylation (G) or ERK phosphorylation (H) was determined by immunoblotting. Shown is a representative of 7 (G) and 5 (H) independently performed experiments and the statistical evaluation (H). Shown are mean values ± S.E.M.; *, $P \leq 0.05$; **, $P \leq 0.01$; ***, $P \leq 0.001$ (one-way ANOVA with Sidak's multiple comparison test (A, C), one-way ANOVA with Tukey's multiple test (B), two-way ANOVA with Sidak's (D) or Tukey's (H) multiple comparison test).

Supplementary Fig. 10

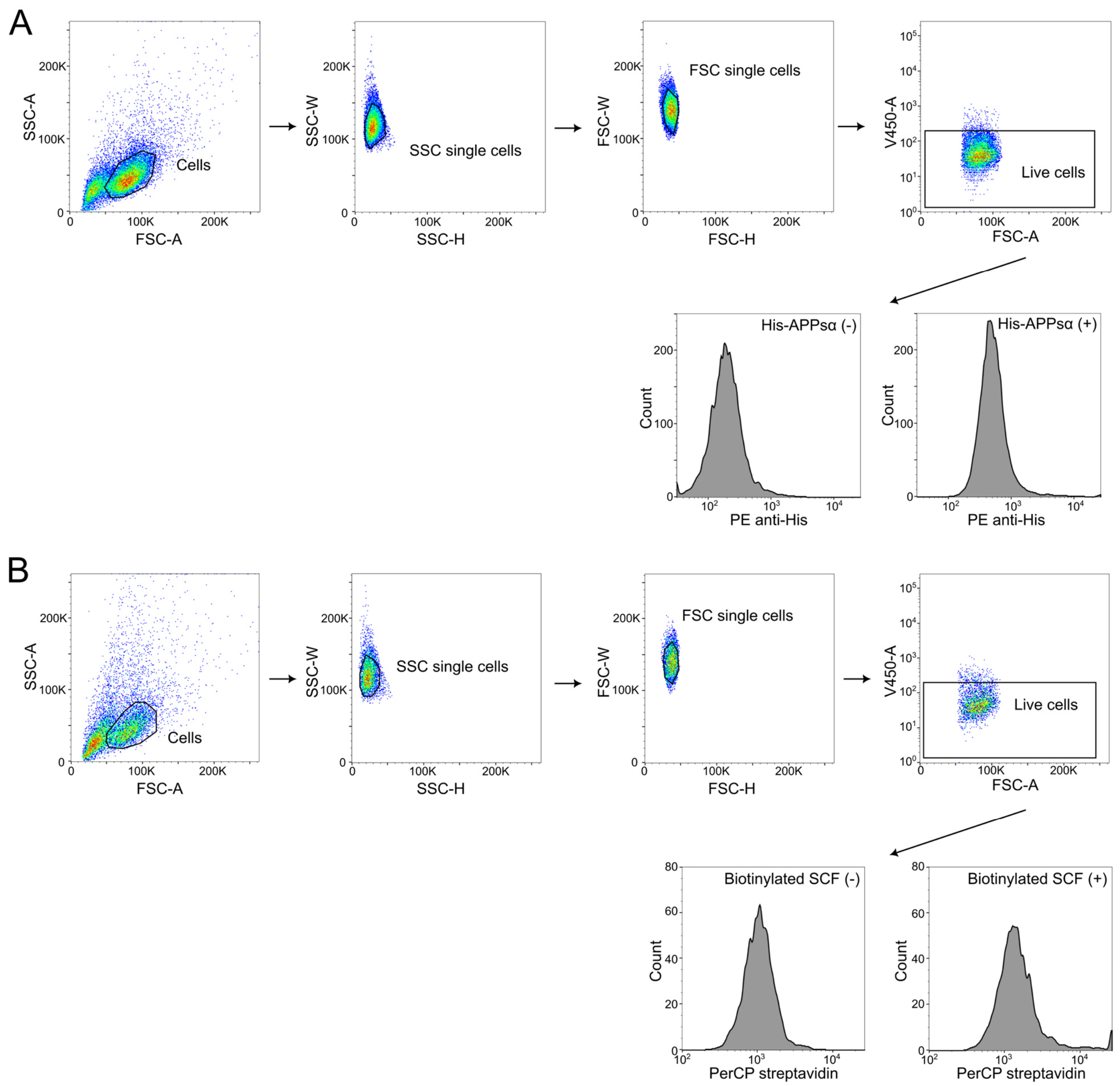

Suppl. Fig. 10. Gating strategy of the binding studies. Binding of His-tagged APPsα to HEK293T cells was determined after staining of cells with phycoerythrin (PE)-conjugated anti-His antibody (A), and binding of biotinylated SCF to HEK293T cells was analyzed after staining of cells with peridinin-chlorophyll-protein (PerCP)-conjugated streptavidin by flow cytometry (B). Cells were first identified based on their size and granularity using a forward-scatter area (FSC-A) versus side-scatter area (SSC-A) plot. Singlet cells were subsequently selected using SSC-H versus SSC-W and FSC-H versus FSC-W plots. From the singlet population, live cells were identified by exclusion of the fraction positive for the dead cell dye. The histograms of the marker of interest, His-PE or streptavidin-PerCP, demonstrate the shift of samples relative to the negative controls.

Supplementary Tables

Suppl. Tab. 1. Targeted sequences of esiRNAs.

Gene	Targeted sequence
APP (human)	CTCGTTCCTGACAAGTGCAAATTCTTACACCAGGAGAGGATGGATGTTTGCGA<br>AACTCATCTTCACTGGCACACCGTCGCCAAAGAGACATGCAGTGAGAAGAGT<br>ACCAACTTGCATGACTACGGCATGTTGCTGCCCTGCGGAATTGACAAGTTCCG<br>AGGGGTAGAGTTTGTGTGTTGCCCACTGGCTGAAGAAAGTGACAATGTGGAT<br>TCTGCTGATGCGGAGGAGGATGACTCGGATGTCTGGTGGGGCGGAGCAGAC<br>ACAGACTATGCAGATGGGAGTGAAGACAAAGTAGTAGAAGTAGCAGAGGAGG<br>AAGAAGTGGCTGAGGTGGAAGAAGAAGAAGCCGATGATGACGAGGACGATG<br>AGGATGGTGATGAGGTAGAGGAAGAGGCTGAGGAACCCTACGAAGAAGCCA<br>CAGAGAGAACCACCAGCA
APLP2 (human)	AAGGAATGGGAAGAGGCAGAGCTTCAAGCTAAGAACCTCCCCAAAGCAGAGA<br>GGCAGACTCTGATTCAGCACTTCCAAGCCATGGTTAAAGCTTTAGAGAAGGAA<br>GCAGCCAGTGAGAAGCAGCAGCTGGTGGAGACCCACCTGGCCCGAGTGGAA<br>GCTATGCTGAATGACCGCCGTCGGATGGCTCTGGAGAACTACCTGGCTGCCT<br>TGCAGTCTGACCCGCCACGGCCTCATCGCATTCTCCAGGCCTTACGGCGTTA<br>TGTCCGTGCTGAGAACAAAGATCGCTTACATACCATCCGTCATTACCAGCATG<br>TGTTGGCTGTTGACCCAGAAAAGGCGGCCCAGATGAAATCCCAGGTGATGAC<br>ACATCTCCACGTGATTGAAGAAAGGAGGAACCAAAGCCTCTC
EGFP	GTGAGCAAGGGCGAGGAGCTGTTCACCGGGGTGGTGCCCATCCTGGTCGAG<br>CTGGACGGCGACGTAAACGGCCACAAGTTCAGCGTGTCCGGCGAGGGCGAG<br>GGCGATGCCACCTACGGCAAGCTGACCCTGAAGTTCATCTGCACCACCGGCA<br>AGCTGCCCGTGCCCTGGCCCACCCTCGTGACCACCCTGACCTACGGCGTGC<br>AGTGCTTCAGCCGCTACCCCGACCACATGAAGCAGCACGACTTCTTCAAGTC<br>CGCCATGCCCGAAGGCTACGTCCAGGAGCGCACCATCTTCTTCAAGGACGAC<br>GGCAACTACAAGACCCGCGCCGAGGTGAAGTTCGAGGGCGACACCCTGGTG<br>AACCGCATCGAGCTGAAGGGCATCGACTTCAAGGAGGACGGCAACATCCTGG<br>GGCACAAGCTGGAGTACAACTACAACAGCCACAACGTCTATATCATGGCCGA<br>CAAGCAGAAGAACGGCATCAAGGTGAACTTCAAGATCCGCCACAACATCGAG<br>GACGGCAGCGTGCAGCTCGCCGACCACTACCAGCAGAACACCCCCATCGGC<br>GACGGCCCCGTGCTGCTGCCCGACAACCACTACCTGAGCACCCAGTCCGCC<br>CTGAGCAAAGACCCCAACGAGAAGCGCGATCACATGGTCCTGCTGGAGTTCG<br>TGACCGCCGCCGGGATCACTCTCGGCATGGACGAGCTGTA

Suppl. Table 2. Sequences of siRNAs.

Gene	Sequence (5' to 3')	siRNA ID	Ref Seq
KIT	GAAAUAUCCUCCUUACUCA	SASI_Hs01_00088058	NM_000222
KIT	GUACUAAUGAGUACAUGGA	SASI_Hs01_00088060	NM_000222
FGFR1	UACGGCAGCAUCAACCACA	SASI_Hs02_00356351	NM_023100

Suppl. Tab. 3. Antibodies used for Western blotting.

**Primary antibody**	**Catalog #**	**Source**	**Dilution**
anti-APP (22C11)	MAB348	Sigma-Aldrich	1:1000
anti-APP (Y188)	ab32136	abcam	1:1000
anti-APLP2	ab140624	abcam	1:4000
anti-APLP2 (D2II) (analysis of recombinant mouse APLP2sα)	171617	Calbiochem	1:1000
anti-APLP2 (analysis of recombinant mouse APLP2sα and APLP2sα-CTFα)	490 011	Synaptic Systems	1:1000
anti-phosho (Tyr1175)-VEGFR2	2478	Cell Signaling Technology	1:1000
anti-VEGFR2	2479	Cell Signaling Technology	1:1000
anti-FGFR1	9740	Cell Signaling Technology	1:1000
anti-phospho (Tyr1173)-EGFR	44-794G	Invitrogen	1:1000
anti-EGFR	4267	Cell Signaling Technology	1:1000
anti-phospho (Tyr568/Tyr570)-KIT	07-806	Sigma-Aldrich	1:1000
anti-phospho (Tyr719)-KIT (mouse)	3391	Cell Signaling Technology	1:1000
anti-KIT	37805	Cell Signaling Technology	1:1000
anti-phospho (Ser473)-Akt	4060	Cell Signaling Technology	1:1000
anti-Akt	9272	Cell Signaling Technology	1:1000
anti-phospho (T202/Y204)-p44/42 MAPK	4370	Cell Signaling Technology	1:1000
anti-p44/42 MAPK	4695	Cell Signaling Technology	1:1000
anti-GAPDH	2118	Cell Signaling Technology	1:1000
anti-α-tubulin	2144	Cell Signaling Technology	1:1000
anti-His tag (analysis of recombinant mouse APLP2sα)	27-4710-01	Cytiva	1:1000
anti-HA tag (C29F4) (analysis of recombinant mouse APLP2sα)	3724	Cell Signaling	1:1000
**Secondary antibody**	**Catalog #**	**Source**	**Dilution**
anti-rabbit IgG, HRP-conjugated	7074S	Cell Signaling Technology	1:10,000
anti-mouse IgG, HRP-conjugated	7076S	Cell Signaling Technology	1:10,000
anti-mouse IgG, HRP-conjugated (analysis of recombinant mouse APLP2sα)	115-035-146	Dianova	1:10,000
anti-rabbit IgG, HRP-conjugated (analysis of recombinant mouse APLP2sα)	711-035-152	Dianova	1:10,000

Suppl. Tab. 4. Plasmid constructs.

Plasmid constructs	Description	VecorBuilder ID
pRP[Exp]-Puro-EF1A>EGFP	Control	VB900088-2255zcg
pRP[Exp]-Puro-EF1A>hKIT[NM_000222.3]	Wild-type human KIT	VB240809-1311dcq
pRP[Exp]-Puro-EF1A>{KIT T417I deltaY418D419}	KIT(T417I, Δ418-419) mutant (SCF-independent dimerization)	VB240809-1314fna
pRP[Exp]-Puro-EF1A>{hKIT(D327A)a980c}	KIT(D327A) mutant	VB241015-1629khf
pRP[Exp]-Puro-EF1A>{hKIT(E329A)a986c}	KIT(E329A) mutant	VB241015-1633gsb
pRP[Exp]-Puro-EF1A>{hKIT(K383A)a1147g,a1148c}	KIT(K383A) mutant	VB241015-1634gpn

pRP[Exp]-Puro-EF1A>{hKIT(R420A)a1258g,g1259c}	KIT(R420A) mutant	VB241015-1637vmq
pRP[Exp]-Puro-EF1A>{hKIT(F433A)t1297g,t1298c}	KIT(F433A)mutant	VB241015-1638nbh
pRP[Exp]-Puro-EF1A>{hKIT(R449A)a1345g,g1346c}	KIT(R449A) mutant	VB241015-1639uqq
pRP[Exp]-Puro-EF1A>{hKIT(Q460A)c1378g,a1379c}	KIT(Q460A) mutant	VB241015-1640mzv
pRP[Exp]-Puro-EF1A>{hKIT(N463A)a1387g,a1388c}	KIT(N463A)mutant	VB241015-1642rjn
pRP[Exp]-Puro-EF1A>{hKIT(K471A)a1411g,a1412c}	KIT(K471A) mutant	VB241015-1643dsc
pRP[Exp]-Puro-EF1A>mKit[NM_001122733.1]	Wild-type mouse KIT	VB241220-1204qkx

Suppl. Tab. 5. Sequences of primers for PCR.

Gene	Forward	Reverse
App (mouse)	5’-AAGCGACAGCGTGGATTC-3’	5’-TCCACCCCACCAGACATC-3’
Aplp2 (mouse)	5’-CAGACAAGGAGATTGTTCACGA-3’	5’-AGTACCAACGAGGCATCACA-3’
Cdh5 (mouse)	5’-AACGAGGACAGCAACTTCAC-3’	5’-TGGCATGCTCCCGATTAAAC-3’
Pecam1 (mouse)	5’-ACTCACGCTGGTGCTCTATG-3’	5’-TGCTGTTGATGGTGAAGGAG-3’
Myh6 (mouse)	5’-CGCATCAAGGAGCTCACC-3’	5’-CCTGCAGCCGCATTAAGT-3’
Ptprc (mouse)	5’-CTGAATACCCGTGGAATGCT-3’	5’-GATTGCTGATGAGGGCAGAC-3’
Pdgfra (mouse)	5’-CTCGTGCTTGGTCGGATTT-3’	5’-TCTTCACAGCCACCTTCATTAC-3’
Adam9 (mouse)	5’-TTTCTCCGGCAGTGAGTACA-3’	5’-GCATTGAAGCTTTCCACACA-3’
Adam10 (mouse)	5’-CAAAGATGATTGCTGCTTCG-3’	5’-CTGTACAGCAGGGTCCTTGAC-3’
Adam17 (mouse)	5’-TGTGGTTATTTAAATGCAGATAGTGA-3’	5’-TCTCTTCACTCGACGAACAAAC-3’
Kit (mouse)	5’-GAGTTCCATAGACTCCAGCGTC-3’	5’-AATGAGCAGCGGCGTGAACAGA-3’
Kitl (mouse)	5’-CTCAACTATGTCGCCGGGAT-3’	5’-CTTCGGTGCGTTTTCTTCCA-3’
Gapdh (mouse)	5’-GGAGAAACCTGCCAAGTATGA-3’	5’-TCCTCAGTGTAGCCCAAGA-3’
Rn18s (mouse)	5’-GCTCTAGAATTACCACAGTTATCCAA-3’	5’-AAATCAGTTATGGTTCCTTTGGTC-3’
ADAM9 (human)	5’-GAGTGTGCATATGGTGACTGTTG-3’	5’-ACTGGTTTTTCCTCGGCATA-3’
ADAM10 (human)	5’-ATATTACGGAACACGAGAAGCTG-3’	5’-TCAATCGCTTTAACATGACTGG-3’
ADAM17 (human)	5’-CCTTTCTGCGAGAGGGAAC-3’	5’-CACCTTGCAGGAGTTGTCAGT-3’
GAPDH (human)	5’-GCATCCTGGGCTACACTGA-3’	5’-CCAGCGTCAAAGGTGGAG-3’
RNA18S1	5’-CCGATTGGATGGTTTAGTGAG-3’	5’-AGTTCGACCGTCTTCTCAGC-3’

(human)		

Suppl. Tab. 6. Antibodies used for analysis of immune cell infiltration in the heart

Antibody	Catalog #	Source	Amount in the cocktail
FITC anti-CD45	553079	BD	1 µl
BV510 anti-Cd11b	101263	Biolegend	1 µl
PerCP-Cy5.5 anti-Ly6G	560602	BD	1 µl
APC-Cy7 anti-Ly6C	560596	BD	1 µl
PE anti-CD64	139303	Biolegend	1 µl
APC anti-CCR2	150627	Biolegend	1 µl
PE Cy7 anti-Tim4	130010	Biolegend	3 µl
PE anti-CD62L	12-0621-82	eBioscience	1 µl (neutrophil panel); 0.125 µl (T-cell panel)
APC anti-CD63	143906	Biolegend	1 µl
APC anti-CD206	141708	Biolegend	1 µl
PeCy7 anti-CD86	105014	Biolegend	1 µl
FITC anti-CD3e	100306	Biolegend	0.5 µl
APC-eFl780 anti-CD4	47-0042-82	eBioscience	0.5 µl
Pe-Cy7 anti-CD8a	25-0081-82	eBioscience	0.17 µl
BV711 anti-CD25	740714	BD	1 µl
Alexa Fluor 700 anti-CD44	103026	Biolegend	0.25 µl
BV421 anti-CD120b	564088	BD	0.25 µl
APC anti-FoxP3	17-5773-82	eBioscience	2 µl
PE/Dazzle™ 594 anti-CD152	106318	Biolegend	2 µl

Suppl. Tab. 7. Antibodies used for immmunohistochemistry and functional studies

**Primary antibody**	**Catalog #**	**Source**	**Dilution**
anti-CD31	550274	BD	1:50
**Secondary antibody**			
Goat anti-Rat IgG (H+L), AlexaFluor™ 488	A-11006	Invitrogen	1:500
**SCF neutralizing antibody**			
anti-SCF	AF-255-NA	R&D	0.3 -1.0 µg/mL
